# Inflammatory response in mouse lungs to haze episodes under different backgrounds of particulate matter exposure

**DOI:** 10.1038/s41598-023-49014-3

**Published:** 2023-12-07

**Authors:** Yuanhang Zhang, Yuteng Zhang, Kai Liu, Ningning Zhu, Jianfeng Pang, Xin Qian, Huiming Li, Xuemei Liu

**Affiliations:** 1 School of Environment, Nanjing Normal University, Nanjing, China; 2 National and Local Joint Engineering Research Center for Deep Utilization Technology of Rock-salt Resource, Huaiyin Institute of Technology, Huaian, China; 3 Jiangsu Collaborative Innovation Center of Atmospheric Environment and Equipment Technology (CICAEET), Nanjing University of Information Science and Technology, Nanjing, China

**Keywords:** Environmental sciences, Biomarkers, Health care

## Abstract

Particulate matter (PM) toxicity has mostly been investigated through in vitro exposure or tracheal infusion in animal models. However, given the complexity of ambient conditions, most animal studies do not mimic real-life PM exposure. In this work, we established a novel integrated exposure model to study the dynamic inflammatory response and defense strategies in ambient PM-exposed mice. Three groups of male C57BL/6 mice were kept in three chambers with pre-exposure to filtered air (FA), unfiltered air (UFA), or the air with a low PM concentration (PM_2.5_ ≤ 75 μg/m^3^) (LPM), respectively, for 37 days. Then all three groups of mice were exposed to haze challenge for 3 days, followed by exposure in filtered air for 7 days to allow recovery. Our results suggest that following a haze challenge, the defense strategies of mice of filtered air (FA) and low PM (LPM) groups comprised a form of “counterattack”, whereas the response of the unfiltered air (UFA) group could be viewed as a “silence”. While the latter strategy protected the lung tissues of mice from acute inflammatory damage, it also foreshadowed the development of chronic inflammatory diseases. These findings contribute to explaining previously documented PM-associated pathogenic mechanisms.

Urban atmospheric pollution arising from rapid economic development in megacities is one of the most serious environmental issues globally^1^. Ambient particulate matter (PM) is an important air pollutant that threatens human health by inducing acute or chronic inflammatory responses and subsequent immune dysregulation^2,3^. PM exposure-induced chronic inflammation has been closely linked with the incidence and development of asthma^4,5^, pulmonary disease^6^, cardiovascular disease^7^, diabetes^8,9^, and Alzheimer's disease^10,11^.

The relationship between PM-induced inflammatory responses and inflammatory diseases has been established, but the regulation of inflammation-mediated protective response is poorly understood. In-depth experimental studies of immune defense mechanisms suggest that some typical “toxic effects” instead protect against or contribute to minimizing the damage caused by PM exposure stress. For example, mild or moderate acute PM exposure may activate macrophages and initiate their cell cycle arrest (thus inhibiting proliferation) or even apoptosis^12–14^. Activated macrophages increase their phagocytic capacity; cell cycle arrest implies damage control at the expense of growth; and apoptotic cells may protect surrounding healthy cells from further damage by preventing the movement of pollutants^15^. Proinflammatory response has been associated with a senescence-like state induced by prolonged PM exposure^16,17^. Senescence followed by clearance and regeneration may ultimately result in the recovery of tissue function (except in aged tissues or in pathological contexts)^18^. These inflammatory responses allow tissues to tolerate stressful conditions, although it is uncertain whether they also mediate subsequent chronic inflammatory diseases arising from prolonged PM exposure. Understanding the pathological effects of PM exposure may be inspired by studying the inflammatory response as a defense strategy combating the adverse effects of PM.

The size distribution, concentration, and chemical composition of PM are the main determinants of toxicity. And all of these characteristics may vary depending on the geographic region as well as the season and the time of day^19^. The complexity of actual PM exposure has been neglected in many animal experiments with in vitro exposure or tracheal infusion, which cannot simulate practical exposures. Several animal experiments have included prolonged ambient PM exposure to study the neurotoxicity^20^, reproductive toxicity^21^, and endocrine toxicity^22^, reporting both staged and final exposure results. However, they neither distinguished the pathological response under alternating PM exposure scenarios nor explored the defense strategies underlying inflammatory response. Haze is a severe pollution phenomenon, characterized by high loading of fine aerosol and atmospheric visibility to < 10 km^23,24^. Hazy weather frequently leads to alternating exposures to high and low PM concentrations, especially in developing countries with high-level of PM pollution. We have found that the dynamic changes of antioxidant defensive response in mice to haze exposure depends on the preliminary PM exposure background^25^. However, the detailed inflammatory response upon alternating PM exposure scenarios remains unclear, hindering the understanding for the immune regulation under these scenarios.

In this work, we established a novel integrated PM exposure model that included pre-exposure, haze challenge, and filtered air recovery stage. Three PM pre-exposure backgrounds were designed to imitate the absence of PM exposure, low PM exposure, or high PM exposure, respectively. Then we analyzed the dynamic inflammatory response and immune defense strategies in mice under complex PM exposure scenarios. Finally, we summarized the possible mechanisms and discussed their significance.

## Results

### Concentrations of PM

**Figure 1:**
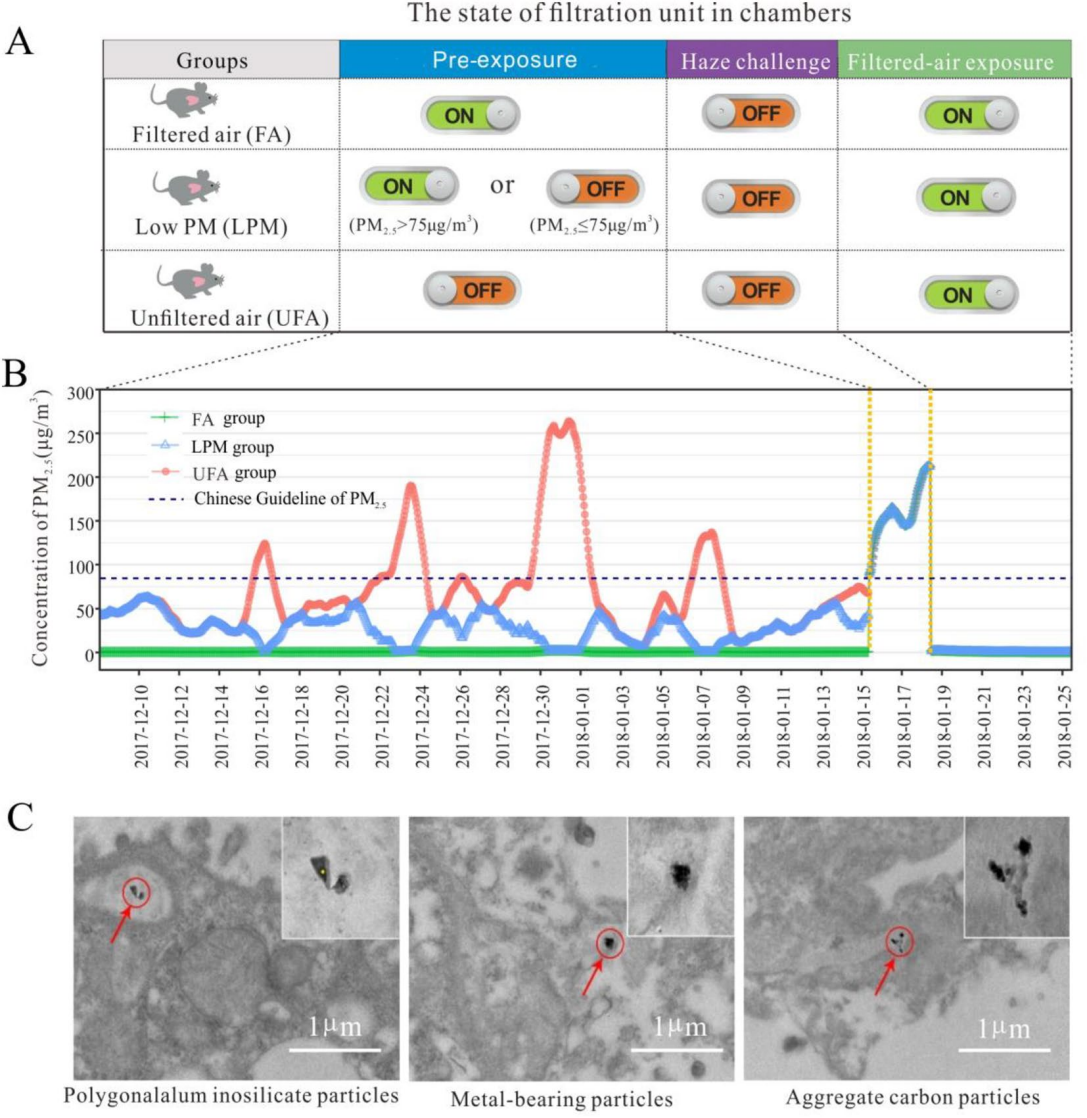
(**A**) The state of filtration unit in chambers during pre-exposure (PE), haze challenge (HC), or filtered air recovery stages. The “ON” and “OFF” buttons represented the on or off state of the filtration unit, respectively. (**B**) Division of groups and the 24 h-moving average concentrations of PM_2.5_ in each group. PM_2.5_ concentrations in the exposure chambers during the entire exposure. (**C**) The three distinct types of PM detected by high-resolution transmission electron microscopy (HR-TEM) in lung tissues.

PM levels in ambient air monitored during exposure were presented in Fig. 1, which can be also found in our previous study^25^. At the pre-exposure stage, the concentrations of PM_2.5_ in the filtered air (FA) chamber (range: 0.3–2.3 μg/m^3^; mean: 1.0 μg/m^3^) were far below that in the unfiltered air (UFA) chamber (range: 6.6–264 μg/m^3^; mean: 70 μg/m^3^). For the low PM (LPM) chamber, since part of ambient air (PM_2.5_ > 75 μg/m^3^) was replaced by filtered air, its PM_2.5_ concentration was controlled to be at lower levels (range: 0.7–63.2 μg/m^3^; mean: 29 μg/m^3^). The PM_2.5_ concentrations in the UFA chamber peaked four times, with maximum concentrations of 121, 187, 261, and 134 μg/m^3^. In the haze challenge stage, all mice in the three groups were subjected to haze pollution with PM_2.5_ concentration ranging from 101 to 246 μg/m^3^. In therecovery stage, the air to all three chambers was filtered to have PM_2.5_ concentrations below 5 μg/m^3^.

Suspected foreign particles in lungs were detected using high-resolution transmission electron microscopy (HR-TEM)/energy-dispersive X-ray spectroscopy (EDS). Delineated particles with clear boundaries were found in lung tissues of mice from the UFA group (Fig. S2 and S3). In contrast, very few suspected foreign particles were observed in the lung tissues of mice in FA and LPM groups. We analyzed the shape, size, and elemental composition of these particles (Fig. S2), suggesting that they included mineral dust particles, metal-bearing particles, and organic carbon particles^26,27^.

### Inflammatory responses induced by three different pre-exposures

**Figure 2:**
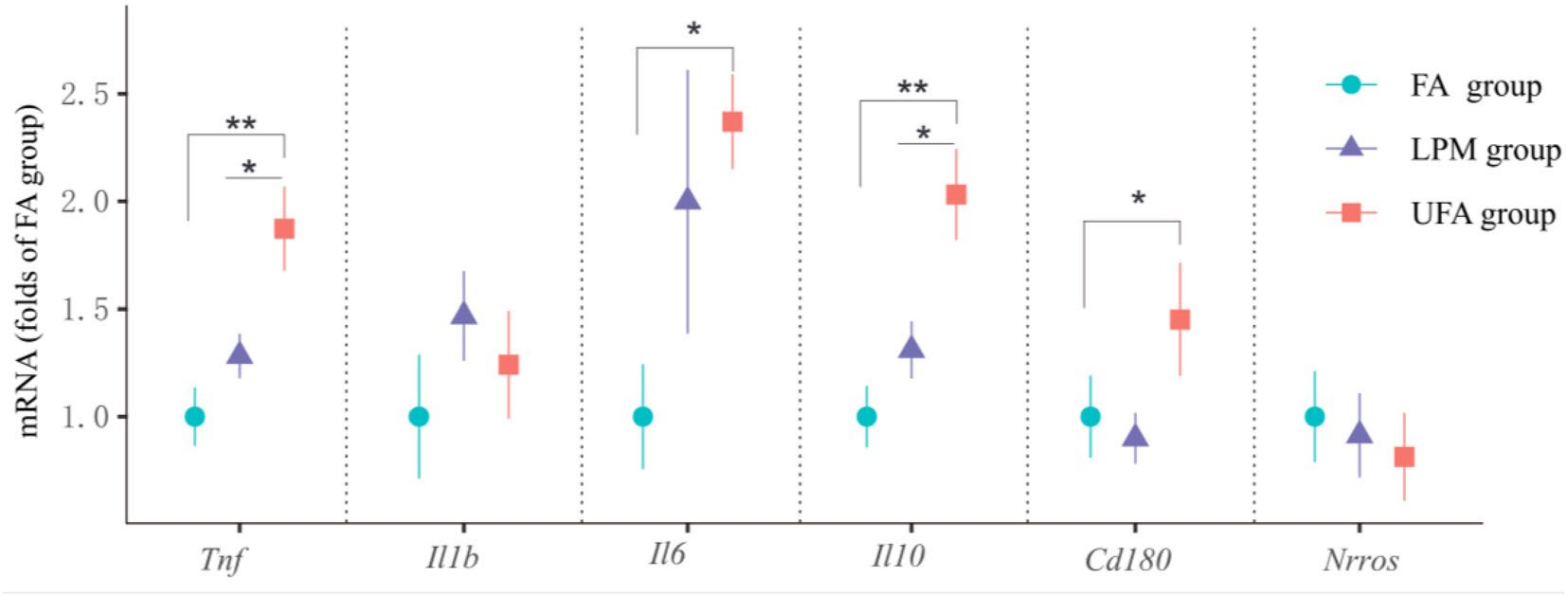
The relative mRNA expression levels of pro- and anti-inflammatory factors at the termination of pre-exposure in mouse lungs with pre-exposure to filtered air (FA), unfiltered air (UFA), or the air with a low PM concentration (LPM), respectively. The bars show the standard error of the mean (n = 5–7); *p < 0.05 and **p < 0.01.

The balance between proinflammatory and anti-inflammatory components determines inflammatory response and thus clinical outcome. Three representative proinflammatory cytokines (tumor necrosis factor, *Tnf*; interleukin 1B, *Il1b*; and interleukin 6, *Il6*) and three anti-inflammatory mediators (interleukin 10, *Il10*; cluster of differentiation 180, *Cd180*; and negative regulator of ROS, *Nrros*) were used as markers to detect inflammatory responses^28–31^. Exposure to ambient air for 37 days remarkably elevated the expression of proinflammatory cytokines (Fig. 2). The expression levels of *Tnf* and *Il6* in the UFA group were 1.87-fold and 2.37-fold higher, respectively, than those in the FA group. For anti-inflammatory factors, the expression levels of *Il10* and *Cd180* in the UFA group were 2.03-fold and 1.45-fold higher, respectively, than those in the FA group. The expression levels of all tested genes didn’t show significant differences between LPM group and FA group (Fig. 2).

### The dynamic proinflammatory response to haze challenge

**Figure 3:**
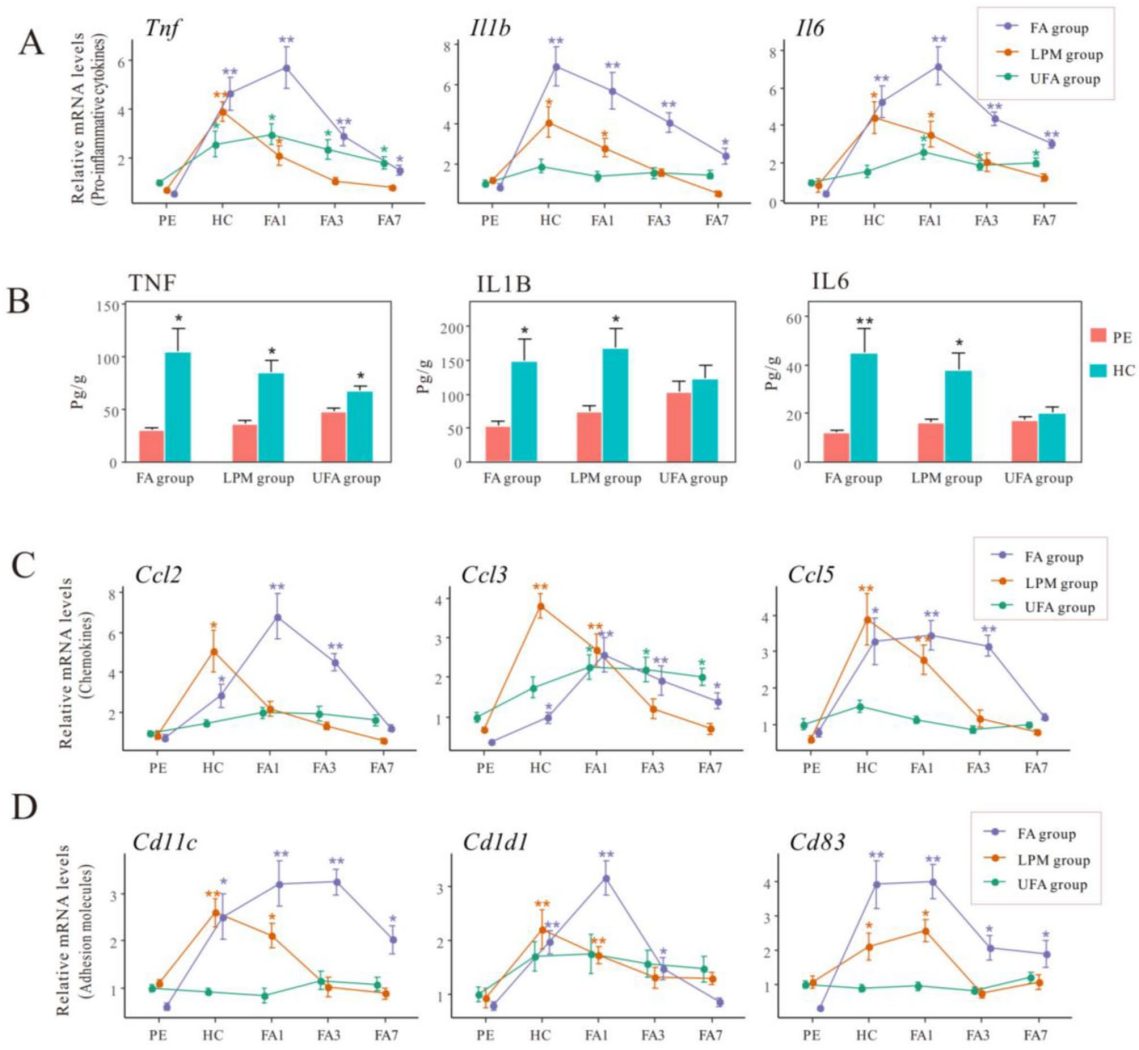
The mRNA levels of the proinflammatory cytokines *Tnf*, *Il1b*, and *Il6* the lungs of mice (**A**), chemokines *Ccl2*, *Ccl3*, and *Ccl5* (**C**), and adhesion molecules *Cd11c*, *Cd1d1*, *and Cd83* (**D**) were measured at the termination of pre-exposure (PE), termination of haze challenge (HC), and on day 1 (FA1), day 3 (FA3), and day 7 (FA7) of the filtered air recovery stage in the three groups with pre-exposure to filtered air (FA), unfiltered air (UFA), or the air with a low PM concentration (LPM), respectively. The protein levels of TNF, IL1B, and IL6 (**B**) were measured during PE and HC stages. The bars show the error of the mean (n = 5–7); *p < 0.05 and **p < 0.01 compared with PE.

The dynamic proinflammatory response was detected by measuring the expression levels of *Tnf*, *Il1b*, and *Il6* at different time points (Fig. 3A; Table S2). Haze challenge significantly enhanced the expression levels of three proinflammatory cytokines in FA and LPM groups. The increase in *Tnf* mRNA levels in the UFA group at the end of haze challenge was much smaller (2.75-fold) than in either FA group (8.72-fold) or LPM group (5.71-fold). Haze challenge failed to induce the expression of *Il1b* and *Il6* in the UFA group. The expression of three proinflammatory cytokines in FA and LPM group began to decline in recovery stage with filtered air. *Tnf* and *Il6* in the UFA group sustained relatively high expression levels in this stage. The expression of selected proinflammatory genes was further validated at protein level (Fig. 3B and Fig. S3). Haze challenge induced remarkable increase in three proinflammatory cytokines in FA and LPM groups compared to UFA group, which was consistent with their changes at transcriptional level.

Chemokines and adhesion molecules are two other important mediators that amplify proinflammatory signals^32^. We detected the expression levels of three chemokines (*Ccl2*, *Ccl3*, and *Ccl5*) and three adhesion molecules (*Cd11c*, *Cd1d1*, and *Cd83*) upon PM exposure (Fig. 3C,D). Haze challenge significantly upregulated the expression of all six genes in FA and LPM groups. In the UFA group, only *Ccl3* was upregulated by haze challenge, maintained high expression level in recovery stage. The expression levels of other five genes in the UFA group didn’t show significant changes upon different exposure stages.

### The dynamic anti-inflammatory response to haze challenge

**Figure 4:**
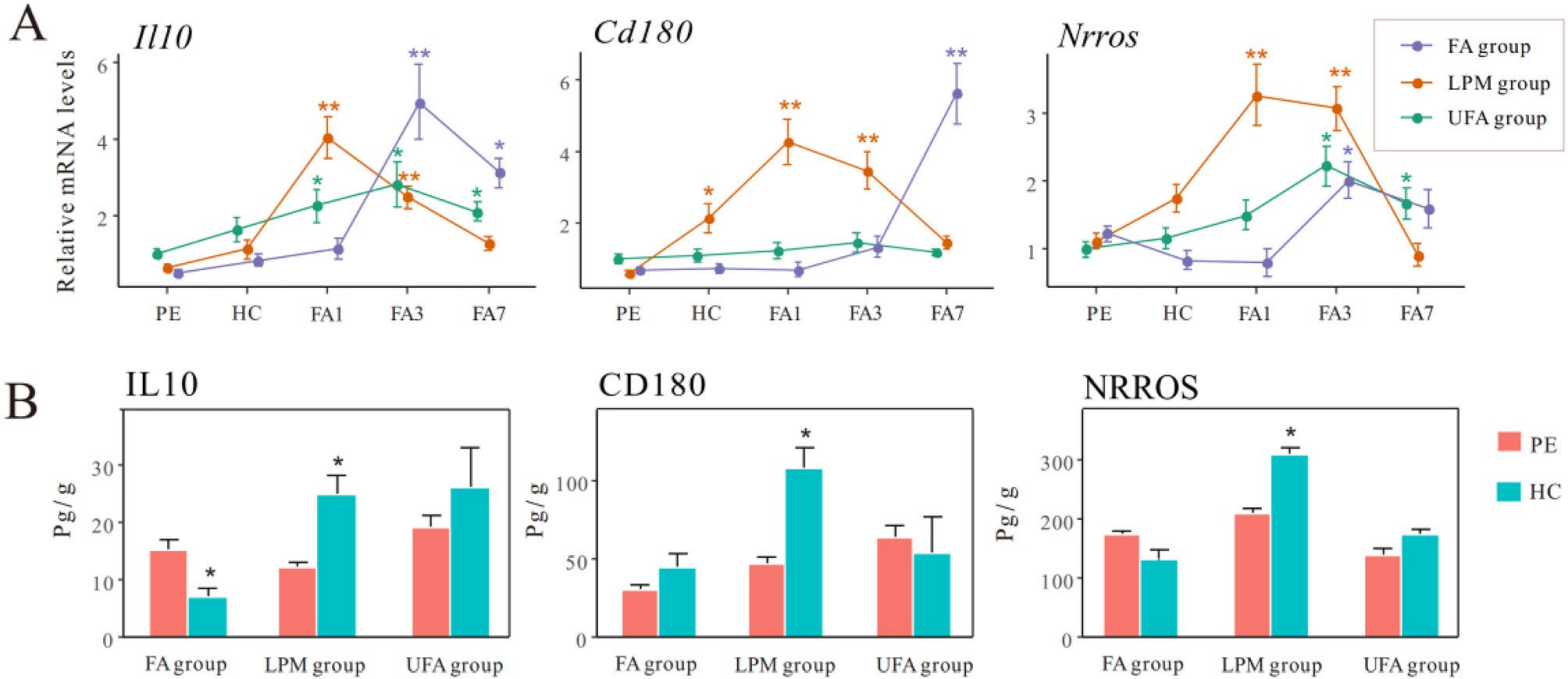
*Il10*, *Cd180*, and *Nrros* mRNA expression in mouse lungs (**A**) was assayed at the termination of pre-exposure (PE), the termination of haze challenge (HC), and on day 1 (FA1), day 3 (FA3), and day 7 (FA7) of the filtered air recovery stage in the three groups with pre-exposure to filtered air (FA), unfiltered air (UFA), or the air with a low PM concentration (LPM), respectively. IL10, CD180, and NRROS protein levels (**B**) were measured in PE and HC. The bars indicate the standard error of the mean (n = 5–7); *p < 0.05 and **p < 0.01 compared with PE.

We analyzed the expression levels of three typical anti-inflammatory mediators *Il10*, *Cd180*, and *Nrros* (Fig. 4A; Table S3). In the FA group, remarkable upregulation of these three genes were only found at the later stage of recovery (FA3 and FA7). An earlier anti-inflammatory response was observed in the LPM group, evidenced by an increase in *Cd180* mRNA level in response to haze challenge and the increase in *Il10* and *Nrros* mRNA levels at FA1. In the UFA group *Il10* and *Nrros* mRNA levels began to increase at FA1 and FA3, respectively, but still smaller than those in the LPM group.

The anti-inflammatory response was further assessed by measuring the protein levels of IL10, CD180, and NRROS (Fig. 4B and Fig. S4). The abundance of all three anti-inflammatory factors in the LPM group significantly increased after haze challenge, but not happened in FA and UFA groups. This was consistent with the changing patterns of anti-inflammatory factors at transcriptional level. These results suggested that anti-inflammatory response was activated timely upon haze challenge in LPM but was delayed in FA and UFA groups.

### Resolution of inflammatory response and damage repair response to haze challenge

**Figure 5:**
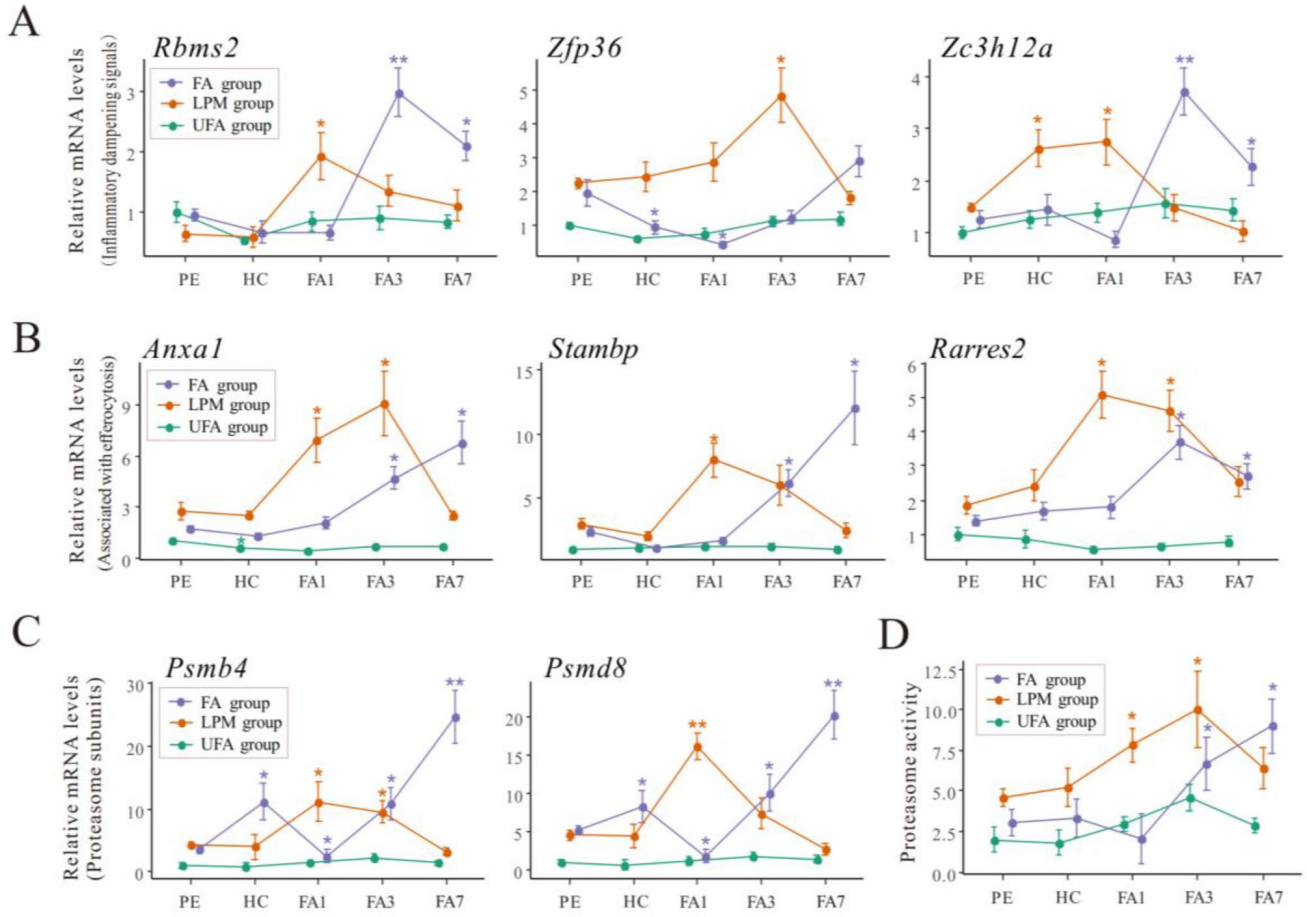
The expression levels of biomarkers associated with dampening of proinflammatory signals (**A**), efferocytosis (**B**), and the degradation of damaged proteins (**C**, **D**) in the mice lungs after haze challenge, as determined at the termination of the pre-exposure (PE) and haze challenge (HC) as well as on day 1 (FA1), day 3 (FA3), and day 7 (FA7) of the filtered air recovery stage with pre-exposure to filtered air (FA), unfiltered air (FA), or the air with a low PM concentration (LPM), respectively. Bars show the standard error of the mean (n = 5–7); *p < 0.05 and **p < 0.01 compared with PE.

The crucial steps in inflammation resolution include dampening proinflammatory signals, eliminating inflammatory cells, and degrading damaged proteins^33^. RNA binding motif, single stranded interacting protein 2 (RBMS2), zinc finger protein 36 (ZFP36), and zinc finger CCCH domain-containing protein 12 (ZC3H12A) are involved in the dampening of proinflammatory signaling^34–36^. We detected the mRNA levels of these three genes upon PM exposure (Fig. 5A; Table S4). The expression levels of *Rbms2*, *Zfp36*, and *Zc3h12a* in the LPM group increased significantly after haze challenge (from HC to FA3) before returning to their initial levels at the end of recovery (FA7). In the FA group, the mRNA levels of *Rbms2* and *Zc3h12a* began to increase at FA3 followed by decreasing at FA7. In the UFA group, different exposures failed to affect the expression of these three genes associated with the resolution of inflammatory response.

Annexin A1 (ANXA1), signal-transducing adaptor molecule (STAM) binding protein (STAMBP), and retinoid acid receptor responder 2 (RARRES2)-dependent signaling pathways play important roles in the clearance of apoptotic inflammatory cells through efferocytosis during inflammation resolution^37–39^. In the LPM group, the mRNA levels of all these three genes began to increase significantly at the beginning of recovery (FA1) followed by declining to their initial levels at the end of recovery (FA7) (Fig. 5B). In the FA group, the mRNA levels of the three genes began to increase significantly at FA3. The expression levels of *Anxa1* and *Stambp* continued to rise, reaching a peak at the end of recovery (FA7), whereas the expression of *Rarres2* declined at FA7 (but still higher than the initial level). For the UFA group, the expression levels of *Anxa1*, *Stambp*, and *Rarres2* were much lower than those in other two groups throughout the exposure process. Different exposures didn’t affect the expression of these three genes, with only a slight decrease in the expression of *Anxa1* upon haze challenge (Fig. 5B).

The biological function of proteasome involves the degradation of damaged proteins and the repair of damaged DNA^40^. The function of proteasome was assessed by measuring the mRNA level of two proteasomal subunits (*Psmb4* and *Psmd8*) and proteasomal activity (Fig. 5C,D). In the LPM group, the mRNA levels of *Psmb4* and *Psmd8* began to increase significantly at FA1 followed by declining to their initial levels at FA7. In the FA group, the early increase in *Psmb4* and *Psmd8* expression occurred at HC followed by a dramatic decline at FA1. Then the mRNA levels of them remarkably increased at FA3, reaching a peak at FA7. Compared with other two groups, the mRNA levels of both genes in the UFA group were significantly lower. The expression of *Psmb4* and *Psmd8* didn’t change significantly throughout the entire exposure process (Fig. 5C). The changes of proteasomal activity showed a similar trend to the expression of *Psmb4* and *Psmd8* in all three groups (Fig. 5D).

### Activated signaling pathways associated with cell cycle

**Figure 6:**
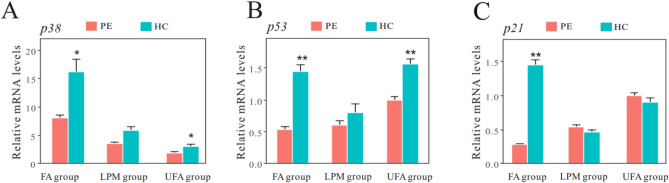
The mRNA expression of *p38* (**A**), *p53* (**B**) and *p21* (**C**) in mouse lungs were assayed at the termination of pre-exposure (PE), the termination of haze challenge (HC) in the three groups with pre-exposure to filtered air (FA), unfiltered air (UFA), or the air with a low PM concentration (LPM), respectively. Bars show the standard error of the mean (n = 5–7). *p < 0.05 and **p < 0.01 compared with PE.

The p53/p21 signaling pathway plays a core role in regulating cell cycle arrest, whereas p38, activated by high intracellular levels of ROS, acts as an important upstream signal of p53/p21 signaling pathway^18^. As shown in Fig. 6, haze challenge significantly upregulated the expression of *p38*, *p53*, and *p21* in the FA group, suggesting the activation of *p38/p53/p21* signaling pathway. However, haze challenge had no effect on the expression of these genes in the LPM group. In the UFA group, haze challenge significantly increased the mRNA levels of *p38* and *p53* mRNA, but not *p21*. However, the mRNA levels of both *p53* and *p21* in the UFA group were consistently high, indicating the activation of cell cycle arrest before haze challenge. The above results demonstrated sustained cell cycle arrest in the UFA group, haze-induced transient cell cycle arrest in the FA group, and silenced cell cycle regulation in the LPM group.

### Histomorphological analysis using H&E and TUNEL staining

**Figure 7:**
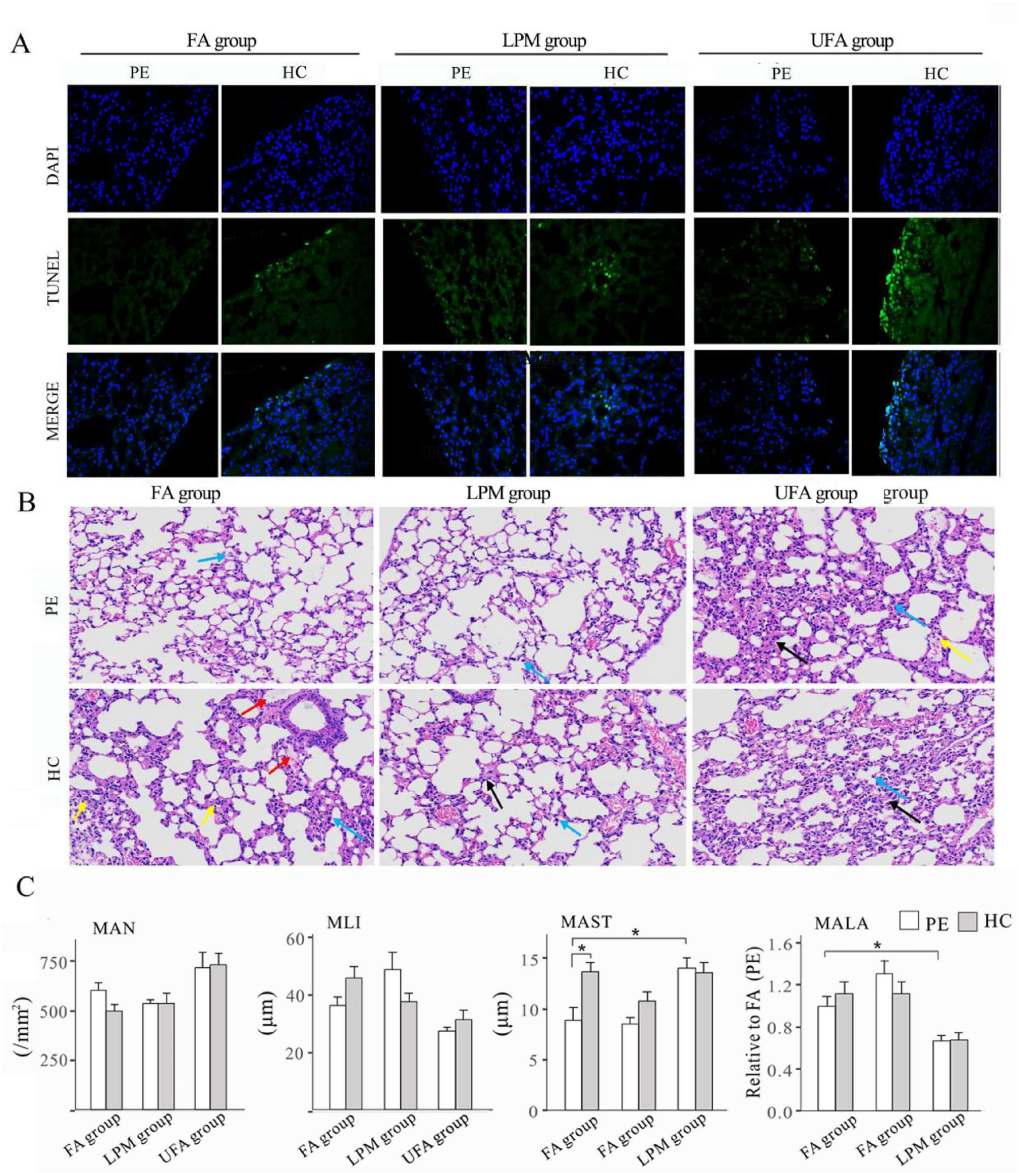
Histomorphological analysis in mouse lungs from the three groups with pre-exposure to filtered air (FA), unfiltered air (UFA), or low PM concentration (LPM), respectively. (**A**) TUNEL analysis of lung tissue of mice at the termination of the pre-exposure (PE) and haze challenge (HE). DAPI-stained nuclei are shown in blue, and the apoptosis signal shows green fluorescence. (**B**) Morphological features of the lung tissues of mice from the three groups at the end of the pre-exposure (PE) and haze challenge (HC). H&E staining, × 200. The black arrows indicate thickened alveolar walls and narrowed alveolar spaces, the blue and yellow arrows indicate infiltrates of macrophages and neutrophils, respectively, and the red arrows indicate pink effusion in the alveolar space. (**C**) The effect of PM exposure on mean alveolar number (MAN), mean linear intercept (MLI), mean alveolar septal thickness (MAST), and alveolar lumen area (MALA) of lung tissues of mice in different groups. Bars show the standard error of the mean (n = 3). *p < 0.05 and **p < 0.01.

The apoptosis in lung tissues before and after haze challenge were evaluated by terminal deoxynucleotidyl transferase-mediated nick end labeling (TUNEL) assay (Fig. 7A). The lung tissues in the UFA group showed strongest TUNEL signals before haze challenge, followed by LPM and FA group. Haze challenge induced increases in TUNEL signals in all three groups, with strongest local signal found in the UFA group.

H&E staining was applied to detect histomorphological changes in lung tissues before and after haze challenge (Fig. 7B). In the FA group, haze challenge induced a mild inflammation, evidenced by increased infiltration of neutrophils and pink exudates in the alveoli of mice. In the LPM group, haze exposure led to slight pathological changes, such as slight thickening of the alveolar wall and alveolar lumen stenosis. In the UFA group, thickened alveolar walls, narrowing of alveolar space (black arrow), and infiltration of a small number of macrophages (blue arrow) and neutrophils (yellow arrow) were observed in the lung tissues before and after haze challenge.

The results of the quantitative lung histomorphologic analysis are shown in Fig. 7C. For comparison, the detection of FA group before haze exposure was chosen as a benchmark. No appreciable effects of PM pre-exposure and haze exposure on the values of mean alveolar number (MAN) and mean linear intercept (MLI) were detected, indicating that ambient PM exposure did not significantly alter the structure of lung. Thickening of the alveolar wall/septa and reduction of alveolar lumen area have been reliable indicators of pulmonary inflammation^41^. The haze-induced mild inflammatory response in the FA group was further confirmed by the increased level of mean alveolar septal thickness (MAST). A similar trend was noticed for the analysis of the MAST in the LPM group, however, no statistical significance was observed. The relatively high level of MAST and reduced MALA in the UFA group before and after haze provided compelling evidence for sustained inflammation in the UFA group, consistent with the increased expression of proinflammatory genes.

In summary, although PM exposure triggered some degree of inflammation, there was no severe morphological lung damage in mice depending on haze exposure in all three groups.

## Discussion

An integrated exposure model which is constituted of pre-exposure, haze challenge, and filtered air recovery was used to dissect immune regulatory strategies and the changes in physiological and pathological inflammatory responses of mice under different PM exposure backgrounds in the present work. A strong and extended proinflammatory response was observed in mouse lungs in the FA group (pre-exposure to filtered air), evidenced by significantly upregulation of inflammatory cytokines and a mild inflammation in histomorphology after haze challenge. This group was also characterized by a delay in the upregulation of genes associated with anti-inflammatory response, inflammation resolution, and damage repair, resulted in a temporary imbalance of immune homeostasis. In the FA group, the increase in proinflammatory cytokines and strong suppression of other immune regulatory factors accompanied the activation of p38/p53/p21 signaling pathway mediating cell cycle arrest^42^. Cell cycle arrest is cytotoxic under internal and external stress conditions, but it also protects mitotic cells. Activation of p38/p53/p21 signaling pathway induces a transient state of growth arrest in which DNA is protected by histones. In this process, energy can be conserved by blocked expression of nonessential genes, while the expression of shock and stress proteins (such as proinflammatory cytokines and antioxidant enzymes) increases^43,44^. In our present results, haze-induced growth arrest persisted for at least 1 day, but immune regulatory genes were upregulated after haze challenge for 3 days. Therefore, cell cycle arrest in the FA group led to a temporary imbalance in homeostasis, but the body of mice still allowed tissue repair to promote potential tissue renewal.

Compared with FA group, the defense strategy in the LPM group showed more efficient immune regulatory response to haze challenge, evidenced by moderate upregulation of proinflammatory genes during haze challenge and timely resolution of inflammatory response thereafter. In addition, haze challenge did not induce the upregulation of *p38*, *p53*, and *p21* in the LPM groups, suggesting that only moderate shock or stress occurred, which was further supported by histomorphometric measurements. These results suggested that the immune system of mice can be "trained" by pre-exposure to LPM in to acclimate to subsequent environmental challenge. Our present results also support the "hygiene hypothesis", which holds that moderate exposure to endotoxin has a benign stimulatory effect on the development of the immune system, as do microbial components and other trace toxins bound by PM^45,46^. Nonetheless, there are growing evidences suggesting that long-term exposure to PM at low concentrations adversely affects health. Two prospective cohort studies from the Netherlands and the United States showed that an about 0.7–1.6 year decline in life expectancy can be attributed to long-term exposure to fine PM at a concentration of 10 μg/m^3^^46,47^. Another large national cohort study of nonimmigrant Canadians suggested that the mortality was closely related to long-term exposure to PM_2.5_ at predominantly low concentrations (mean, 8.7 μg/m^3^)^48^. Therefore, other adverse effects on health should be considered upon exposure to low PM concentrations. Further studies aimed at identifying the toxic boundary concentrations of low PM exposure will contribute to a better understanding of the pathogenic mechanisms for PM exposure.

As evident from the heatmap of Fig. S5, the inflammatory response of mice in the UFA group was much lower and narrower than that of in FA and LPM group. Mice in the UFA group had a higher initial level of inflammatory response after pre-exposure, but most of the genes related to inflammatory response didn’t respond to subsequent haze challenge. This suggested that pre-exposure with ambient air had an immunosuppressive effect on mice, which further hindered the response to haze challenge. It has been found that pre-exposure to PM suppresses immune defense abilities and undermines host immune responses to a subsequent infection. For example, mice intratracheally instilled with PM seemed to be more susceptible than non-exposed mice to the subsequent infection of bacterials^49^. Mice with suppression of Th2 responses are exposed to combustion-derived particulate matter in early life, resulting in an immunosuppressive environment in the lung^50^. The harmful effects caused by urban PM pre-exposure are also evidenced by the significantly increased incidence of diseases (such as pneumonia and associated mortality in populations) with further haze exposure^23,51^. However, little is known about the mechanism for immunosuppression induced by PM exposure.

We speculated three possible explanations for the immunosuppression characterized in the UFA group based on our present results combined with previous reports. First, mice in the UFA group may deploy anti-inflammatory compensatory mechanism before haze challenge, thus protected their lungs from excessive inflammatory damage. However, the increased expression of anti-inflammatory genes may inhibit the immune response to subsequent exogenous stimuli or stress^33,52^. Second, haze challenge failed to upregulate the genes encoding for chemokines and adhesion molecules. These genes are involved in inflammatory response-related signaling pathways responsible for antigen presentation and the enhancement of immune cell efficiency^53,54^. Therefore, PM-induced impairment of these pathways may contribute to the low grade inflammatory response determined in mice in the UFA group. Third, cell cycle arrest proteins (p53 and p21) showed high mRNA levels before haze challenge, suggesting that pre-exposure with ambient air induced a growth-arrested state in mice lungs. This kind of "senescence-like" state seemed to be maintained^55,56^, which was different from the transient growth arrest in the FA group. Indeed, several other studies have reported the similar findings of a "senescence-like" status after PM exposure^16,57^. The "senescence-like" status is characterized by a reduced responsiveness to external stimuli and the loss of certain cellular functions^58,59^. Our study demonstrated that the "senescence-like" state in the UFA group accompanied less effective or absent resolution and damage repair. This may explain why the slight inflammatory response cannot be weakened even after transferring to filtered air in the UFA group.

**Figure 8:**
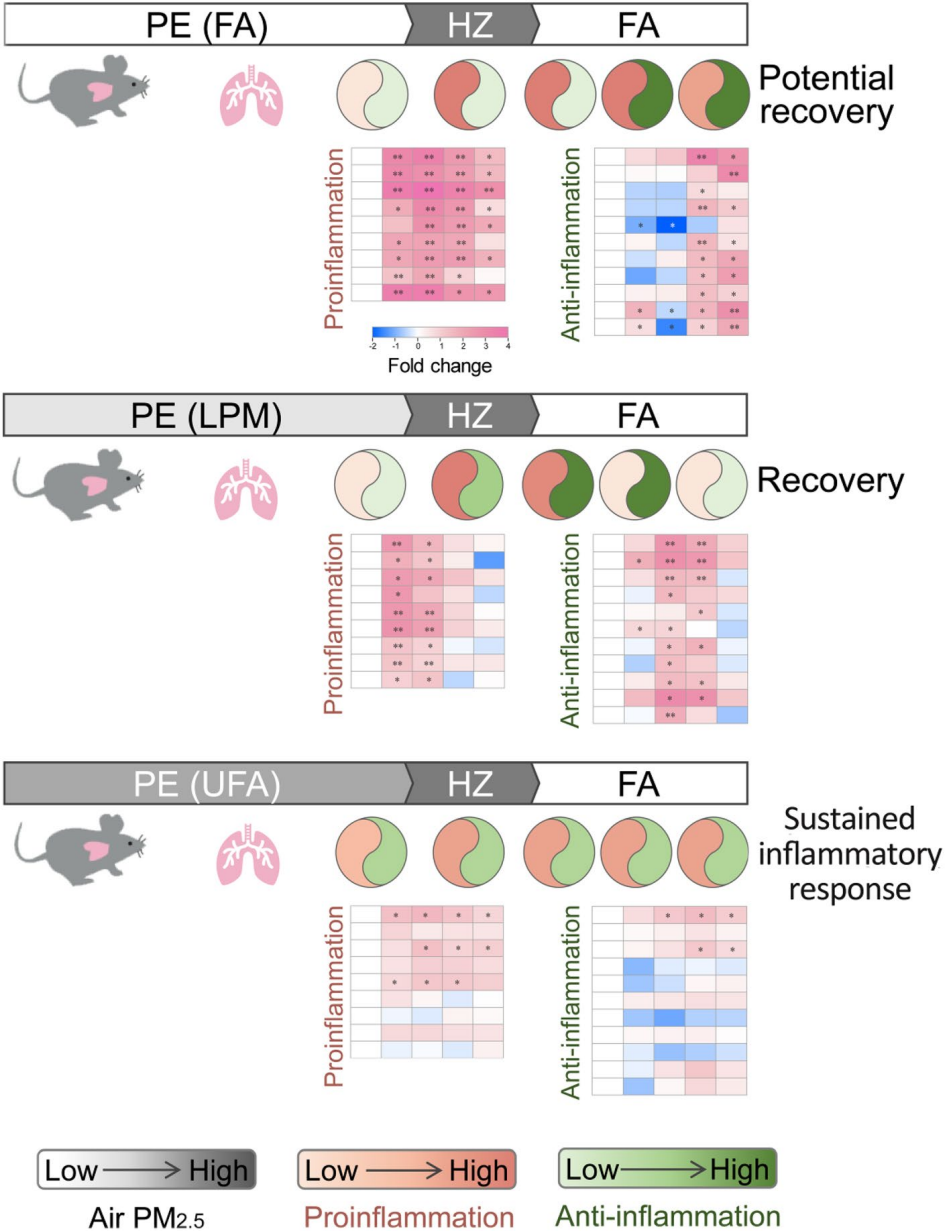
Schematic model of proinflammatory and anti-inflammatory responses in mice lungs upon different PM exposure modes.

We proposed a schematic model summarizing the inflammatory responses of lungs in mice to different PM exposure modes (Fig. 8). Pre-exposure with FA did not affect proinflammatory and anti-inflammatory responses in lungs. Then sudden exposure to haze (HZ) resulted in acute proinflammatory responses, which continued even after transferring to FA environment. At the later stage of FA exposure, anti-inflammatory responses began to increase remarkably while the proinflammatory responses began to decrease. This may facilitate the potential recovery of lungs from inflammatory injury at final FA environment. In this exposure mode (FA → HZ → FA), the inflammatory response is strong but potential reversible. Pre-exposure with LPM is not sufficient to elicit proinflammatory and anti-inflammatory responses in lungs. Further exposure to HZ induced moderate proinflammatory and anti-inflammatory responses in succession, and both of them returned to the initial level at the end of FA stage. In this exposure mode (LPM → HZ → FA), pre-exposure with LPM may help mouse acclimate the PM environment, coping with haze-induced adverse effects more timely and efficiently. Pre-exposure with UFA stimulated both proinflammatory and anti-inflammatory responses in lungs. Further exposure to HZ did not change the inflammatory response significantly except for small increases in a few proinflammatory mediators. Followed exposure to FA slightly increased anti-inflammatory responses, which was not enough to counteract the increased proinflammatory responses. In this exposure mode (UFA → HZ → FA), continuous exposure to UFA suppressed both the inflammatory response capacity and inflammatory resolution function, which led to the mild inflammatory response remained until the end of FA stage. In sum, direct short exposure to heavy PM led to acute inflammatory response in the lungs of mouse, which could be potentially recovered after clean air exposure. Mild PM exposure could help mouse acclimate to the following heavy PM environment in order to avoid inflammatory injury in lungs. Repeated exposure to heavy PM may result in low-level and sustained inflammatory response in the lungs of the mouse.

If the defense strategies that cope with a haze challenge comprised a "counterattack" (demonstrated by the results obtained in the FA and LPM group), the defense strategy in the UFA group can be viewed as a "silence" following a haze challenge. Similar trend has also been observed in our previous study focusing on PM-induced oxidative stress in mice, in which haze exposure fails to affect the expression of most antioxidant defense genes and damage repair function genes in the UFA group (Fig. S6). The suppressed inflammatory response and the compromised redox-stress response in the UFA group may deduce a shared mechanism that is related to PM-induced senescence-like state^60^. Senescent cells tend to be proinflammatory, inducing a response referred to the senescence-associated secretory phenotype (clearance of senescent cells)^61^. However, the process of senescence clearance regeneration may be prematurely terminated by diverse environmental stimuli, including heavy PM. The accumulation of senescent cells may cause chronic inflammation or associated diseases. In a shorter term, the silent response of inflammation and oxidative stress to haze exposure may spare mice from severe inflammatory or oxidative damage. However, in long-term outcome, it would foreshadow chronic inflammation and chronic inflammatory diseases associated with prolonged PM exposure. Indeed, a recent study by Hill et al. demonstrated that the sustained inflammation induced by PM exposure is a "catalyst" for the development of cancer and inhibition of proinflammatory response has been shown to reduce lung cancer incidences, which is consistent with our inference^62^.

Actual ambient air exposure has a great advantage in providing real ambient air exposure situations, but it is almost impossible to precisely control the levels and fluctuation of PM in the chambers of UFA group and LPM group. The randomness could not be entirely controlled in real ambient air exposure experiment, but the results of this study provide preliminary but promising insights into the health effects of urban PM exposure. Further research is needed to identify inflammatory responses representative of beneficial trade-offs in a broader PM exposure context.

## Methods

### Exposure models

Ninety healthy SPF-grade C57BL/6 male mice aged 6 to 8 weeks were randomly divided into three groups (n = 30 each group). The mice in three exposure chambers (1.5 × 0.8 × 0.8 m) were raised placed separately in vinyl sheds. A draught fan placed outdoors (approximately 3 m distance from the shed) was linked to each exposure chamber to allow the ambient air flow in. A high efficiency particulate air (HEPA) filtration was fixed between the air inlet and exposure space of each chamber. The HEPA filter had a particle removal capacity of at least 99.97% for particles with a diameter of 0.3 µm, which includes dust, smoke, pollen, pet dander, and other allergens. Ambient or filtered air was delivered to each exposure chamber by controlling the on/off state of the air filtration unit. All three chambers were ventilated with filtered air for 1 week prior to initial exposure to help all the mice acclimate to the experimental conditions. The experimental shed was built at Nanjing University, located in the northern suburb of the city. Nanjing is a comprehensive industrial production base and an transportation hub in China's Yangtze River Delta region.

The filtration unit of the chamber containing the unfiltered air (UFA) group was switched off to allow the continuous entry of ambient air during the pre-exposure. For the low PM concentration (LPM) group, the filtration unit was switched on once the monitored concentrations of PM_2.5_ (atmospheric particles with an aerodynamic diameter of ≤ 2.5 μm) were beyond the 24-h standard limit for PM_2.5_ (75 μg/m^3^) of China, thus ensuring that mice in this chamber were exposed to only a relatively lower level of PM_2.5_. For the filtered air (FA) group, the filtration unit of the chamber ran continuously during the pre-exposure stage, thus exposing the mice only to filtered air. After 37 days of pre-exposure, Nanjing city experienced a severe haze pollution, at which point the experiment entered the haze exposure stage. During this time, filtration units in all three groups were turned off so that all mice were exposed to haze episodes for 3 days. Then the filtration units were opened, exposing the mice to a nearly PM-free environment for 7 days for recovery. Lung samples of all mice in three groups were obtained at the end of the pre-exposure period, the end of the haze challenge, and on days 1, 3, and 7 during the recovery phase with filtered air. The design of the exposure scheme can be found in Fig. S1. Details of the filtration unit and the selection of exposure time interval are described in Supplemental Materials.

The PM concentration in three chambers during the exposure was monitored using three Dust Mate environmental monitors (Turnkey Instruments Ltd., UK). The experimental conditions consisted of a temperature of 21 ± 4 °C with a continuous 12 h light/dark cycle. Mouse food, drinking water and bedding materials were replaced every 3 days. Cages, chambers, and sheds were disinfected at least once a week. The humane treatment of the mice was a priority. All experimental procedures were approved by the Model Animal Research Center of Nanjing University.

### Preparation of lung tissue samples

Five to seven mice from each group were randomly taken from the exposure environment and euthanized by cervical dislocation at each time point. Both lungs of each mouse were quickly separated by dissection. Then the lungs were washed with ice-cold phosphate-buffered saline. One lobe of lung tissue was divided into two parts: one was directly fixed with 4% paraformaldehyde for light microscopy, while the other was cut into 1 mm^3^ blocks and then fixed in 2.5% glutaraldehyde for high-resolution transmission electron microscopy (HR-TEM). The other lobe was chopped into pieces and divided into five portions for other analysis.

### qRT-PCR

Total RNA was isolated from mice lungs using TRIzol reagent (Invitrogen, USA). A first-stand cDNA synthesis kit (Fermentas, Lithuania) was used to synthesize cDNA from mRNA. Then the cDNA was served as the template to perform quantitative reverse transcription polymerase chain reaction (qRT-PCR) by using Applied Biosystems StepOnePlus™ real-time PCR system (Life Technologies™, USA). The qRT-PCR conditions were as follows: 95 °C for 10 min, followed by 40 cycles of 95 °C for 15 s and 60 °C for 1 min. The data were analyzed using PCR system software based on the 2^−ΔΔCT^ threshold cycle method. Glyceraldehyde-3-phosphate dehydrogenase (GAPDH) was used as a housekeeping gene to normalize the data. The qRT-PCR assays were performed in triplicate. The selection of the indicator genes associated with immune regulation is described in Supplemental Materials. Primer 5.0 was used for the gene amplification of primers (Table S1).

### ELISA analysis

The protein levels of tumor necrosis factor (TNF), interleukin 1B (IL1B), interleukin 6 (IL6), interleukin 10 (IL10), cluster of differentiation 180 (CD180), and leucine-rich repeat-containing protein 33 (NRROS) in the lung were analyzed using commercially available standard sandwich enzyme-linked assay kits (Bioswamp Life Science, Shanghai, China).

### Histomorphological analysis and the detection of foreign particles

Terminal deoxynucleotidyl transferase-mediated nick end labeling (TUNEL) staining was conducted by a TUNEL apoptosis assay kit (Beyotime, C1088). TUNEL-positive cells were imaged using an EVO fluorescence microscope (AMG). Apoptotic cells were those with green fluorescence. Tissue sections with a thickness of 0.5 μm were paraffin-embedded, stained with hematoxylin–eosin (H&E), and observed by optical microscopy (Nikon Eclipse E100, Japan). Base on H&E staining, the following data was measured: (1) mean alveolar number (MAN); (2) mean linear intercept (MLI); (2) mean alveolar septal thickness (MAST); (4) and mean alveolar lumen area (MALA). The determination methods refer to previous literatures^62^.

Lung tissue cut into 60 to 80 nm ultrathin sections was used in the detection of suspected foreign particles by HR-TEM using an FEI Tecnai F20 TEM instrument equipped with energy-dispersive X-ray spectroscopy (EDS).

### Statistical analysis

Two-way ANOVA was used to analyze the between-group differences in the indicators of interest among groups at the predefined time points, in which group and time were the two factors. One-way ANOVA followed by a Tukey post-hoc test was used to compare the intra-group effects of the haze challenge at the five time points, and a two-tailed t-test was used to compute the difference between the two mean values. All statistical analyses were performed using SPSS software, version 23. For plot drawing, R software (R Core Team, New Zealand) was used with ggplot2 and base plot packages. The heatmaps representing relative gene expression level were generated by using TBtools.

### Ethics declaration

This study was conducted in accordance with the ARRIVE guidelines and all relevant ethical regulations. The animal care and use protocols were in strict accordance to the Regulation for Management of Laboratory Animals (1988) and Guidelines for Care and Use of Laboratory Animals (2006) in China. The animal experiments were approved by the Institutional Animal Care and Use Committee of the Model Animal Research Center of Nanjing University (MARC-NJU).

### Supplementary Information

Supplementary Information.

## Data Availability

The datasets used and/or analyzed during the current study are available from the corresponding author on reasonable request.
