# Supplementary material for: Inflammatory response in mouse lungs to haze episodes under different backgrounds of particulate matter exposure
Source: Sci Rep. 2023 Dec 7;13:21616. doi: 10.1038/s41598-023-49014-3 (PMC10703782; doi:10.1038/s41598-023-49014-3)
Supplement: Supplementary file 1 — Supplementary Information. [file 41598_2023_49014_MOESM1_ESM.docx]

**Supplementary Information**

**Inflammatory response in mouse lungs to haze episodes under different backgrounds of particulate matter exposure**

**Yuanhang Zhang ^a,1^, Yuteng Zhang ^a,1^, Kai Liu ^a^, Huan He ^a^, Ningning Zhu ^b^, Jianfeng Pang ^b^, Xin Qian ^c^, Huiming Li ^a,*^, Xuemei Liu ^b,*^**

^a^ School of Environment, Nanjing Normal University, Nanjing, China

^b^ National & Local Joint Engineering Research Center for Deep Utilization Technology of Rock-salt Resource, Huaiyin Institute of Technology, Huaian, China

^c^ Jiangsu Collaborative Innovation Center of Atmospheric Environment and Equipment Technology (CICAEET), Nanjing University of Information Science & Technology, Nanjing, China

**Correspondence**

Xuemei Liu, School of Chemical Engineering, Huaiyin Institute of Technology, E-mail: liuxm7826@163.com

Huiming Li, School of Environment, Nanjing Normal University, E-mail: valen222@126.com

^1^ These authors contribute equally to this work

Contents

[**Detailed Information of the Filtration Unit** 4](#_Toc145537895)

[**Selection Method of the Sampling Time Interval** 4](#_Toc145537896)

[**Sampling Site and PM_2.5_ Sample Preparation** 5](#_Toc145537897)

[**Table S1.** Sequences of the primers used in the quantitative reverse transcription polymerase chain reaction. 8](#_Toc145537898)

[**Table S2.** The mRNA expression levels of proinflammatory cytokines (*Tnf*, *Il1b*, *Il6*), chemokines (*Ccl2*, *Ccl3*, *Ccl5*) and adhesion molecules (*Cd11c*, *Cd1d1*, *Cd180*) at the termination of the preliminary exposure (PE) and haze challenge (HC) phases as well as on day 1 (FA1), day 3 (FA3), and day 7 (FA7) during the filtered air recovery phrase in the three groups with preliminary exposure to filtered air (FA), unfiltered air (UFA), or the air with a low PM concentration (LPM), respectively. 10](#_Toc145537899)

[**Table S3.** Relative mRNA expression levels of anti-inflammatory factors (*Il10*, *Cd180*, *Nrros*) at the termination of the preliminary exposure (PE) and haze challenge (HC) phases as well as on day 1 (FA1), day 3 (FA3) and day 7 (FA7) during the filtered air recovery phase in the three groups with preliminary exposure to filtered air (FA), unfiltered air (UFA), or the air with a low PM concentration (LPM), respectively. 11](#_Toc145537900)

[**Table S4.** Relative mRNA expression levels of genes encoding proteins that dampen proinflammatory signaling (*Rbms2*, *Zfp36*, *Zc3h12a*), participate in efferocytosis (*Anxa1*, *Stambp*, *Rarres2*) and contribute to proteolytic systems (*PMSB4*, *PMSD8*) at the termination of the preliminary exposure (PE) and haze challenge (HC) phases as well as on day 1 (FA1), day 3 (FA3) and day 7 (FA7) during the filtered air recovery phase in the three groups with preliminary exposure to filtered air (FA), unfiltered air (UFA), or the air with a low PM concentration (LPM), respectively. 12](#_Toc145537901)

[**Table S5.** Relative levels of *P38*, *P53* and *P21* mRNA expression at the termination of the preliminary exposure (PE) and haze exposure (HE) phases in the three groups with preliminary exposure to filtered air (FA), unfiltered air (UFA), or the air with a low PM concentration (LPM), respectively. 13](#_Toc145537902)

[**Figure S1.** Scheme for the operating principle of the exposure chamber. The mice were raised in exposure chambers placed separately in vinyl sheds. (A) A draught fan placed outdoors was linked to each exposure chamber to allow the ambient air to flow in. A PM filtration unit was fixed between the air inlet and exposure space in each chamber. (B) Ambient or filtered air was delivered to each exposure chamber by controlling the on/off state of the air filtration unit. 14](#_Toc145537903)

[**Figure S2.** The combined high-resolution transmission electron micrographs and EDS analysis of the lungs of mice in the unfiltered group revealed three distinct types of PM. (A) Polygonal, angular particles containing high levels of silicon (Si), oxygen(O), aluminum (Al), etc., consistent with their MD origin^16^, (B) roughly spherical particles containing heavy metals such as iron (Fe) and chromium (Cr), suggesting the high-temperature formation of nanospheres via combustion and/or friction^17^ and (C) aggregate carbon particles containing high contents of carbon (C) and oxygen (O), identified the presence of fly ash from industrial coal combustion^16^. 15](#_Toc145537904)

[**Figure S3.** The protein levels of TNF, IL1B, and IL6 in mouse lungs were measured at the termination of the preliminary exposure (PE) and haze challenge (HC) phases as well as on day 1 (FA1), day 3 (FA3) and day 7 (FA7) during the filtered air recovery phase in the three groups with preliminary exposure to filtered air (FA), unfiltered air (UFA), or the air with a low PM concentration (LPM), respectively. The bars indicate the error of the mean (n = 5-7); *p < 0.05 and **p < 0.0, compared with the PE time point in a One-way ANOVA followed by Tukey's post-hoc test. 16](#_Toc145537905)

[**Figure S4.** IL10, CD180, and NRROS in mouse lungs protein levels were assayed at the termination of preliminary exposure (PE) and haze challenge (HC) phases as well as on day 1 (FA1), day 3 (FA3) and day 7 (FA7) during the filtered air recovery phase in the three groups with preliminary exposure to filtered air (FA), unfiltered air (UFA), or the air with a low PM concentration (LPM), respectively. The bars indicate the standard error of the mean (n = 5-7); *p < 0.05 and **p < 0.01 compared with the PE time point in a One-way ANOVA followed by Tukey's post-hoc test. 17](#_Toc145537906)

[**Figure S5.** Summarized relative expression level of proinflammatory and anti-inflammatory genes in mouse lungs upon PM exposure. (A) Pre-exposure (PE) with unfiltered air (UFA) followed by haze challenge (HZ) and filtered air (FA) exposure. (B) PE with low PM concentration air (LPM) followed by HZ and FA exposure. (C) PE with FA followed by HZ and FA exposure. The relative expression of each gene at HC and followed FA (FA1, 3, 7) were presented with respect to its expression level at the termination of PE. Relative expression were presented as Log2 fold change against PE treatment. p < 0.05 and **p < 0.01 compared with the PE time point in a One-way ANOVA followed by Tukey's post-hoc test. 18](#_Toc145537907)

[**Figure S6.** Summarized relative expression level of antioxidant-related genes genes in mouse lungs upon PM exposure. (A) Pre-exposure (PE) with unfiltered air (UFA) followed by haze challenge (HZ) and filtered air (FA) exposure. (B) PE with low PM concentration air (LPM) followed by HZ and FA exposure. (C) PE with FA followed by HZ and FA exposure. The relative expression of each gene at HC and followed FA (FA1, 3, 7) were presented with respect to its expression level at the termination of PE. Relative expression were presented as Log2 fold change against PE treatment. p < 0.05 and **p < 0.01 compared with the PE time point in a One-way ANOVA followed by Tukey's post-hoc test. 19](#_Toc145537908)

[**References** 20](#_Toc145537909)

**Detailed Information of the Filtration Unit**

The filtration unit was composed of a HEPA filter (39 cm × 27 cm × 2 cm), a pneumatic sensing system and a DustMate environmental monitor (Turnkey Instruments Ltd., UK). When the filtration unit was off, the HEPA filter was beneath the air inlet, but when the filtration was switched on, it rose and blocked the air inlet. The on/off state of the filtration unit was controlled either manually or by a pneumatic sensing system according to signals generated by the PM_2.5_ concentration coming from a DustMate. In the unfiltered air (UFA) group chamber, the filtration unit was switched off such that ambient air was provided during the entire preliminary exposure phase. In the low PM concentration (LPM) group chamber, the filtration unit was controlled automatically by a signal transducer and was switched on only when the PM_2.5_ concentration exceeded 75 μg/m^3^ (Chinese Guideline for PM_2.5_). This ensured that mice in this chamber were exposed only to ambient air with a low level of PM_2.5_ pollution. In the filtered air (FA) group chamber, the filtration unit was switched on during the entire preliminary exposure phase, such that the mice were exposed only to filtered, very clean air.

The HEPA filter had a particle removal capacity of at least 99.97% for particles with a diameter of 0.3 µm, which includes dust, smoke, pollen, pet dander, and other allergens. A test run showed no obvious differences in the PM_2.5_ concentrations at different positions of the same chamber. The differences in the PM_2.5_ concentration between the unfiltered air in the chamber and the ambient air were ≤ 5 μg/m^3^. The airspace between the filter fibers was more than 0.3 μm, while the gas molecules were usually a few nanometers in diameter. Meanwhile, there are no catalysts for removing gaseous molecules. We monitored the concentrations of gaseous pollutants such as SO_2_, NO_2_, CO and O_3_ in the chambers before the experiment by portable gas pollutant monitoring, and no difference was found among the three chambers. Therefore, it is clear that the only major difference was the PM concentrations among the three chambers.

**Selection Method of the Sampling Time Interval**

Because this is a first attempt to explore the defense strategy to haze challenge under different PM exposure backgrounds, the selection of sampling time interval is crucial to study the levels of proinflammatory and anti-inflammatory markers, indicators of inflammatory resolution and damage repair. In one of our previous experiments, mice in the UFA group exhibited a moderate inflammatory response after exposure to ambient air for 4 and 8 weeks and then exhibited obvious tissue damage after 12 weeks of ambient air exposure^1,2^. Therefore, it is appropriate to set preliminary exposure periods as 4 to 8 weeks. That is, after 4-8 weeks of preliminary exposure, once heavy haze pollution occurs, it is reasonable to make the experiment enter the haze challenge stage. Fortunately, a serious haze episode occurred in Nanjing from January 15 to 19, 2018, just after 5 weeks of preliminary exposure. It is known that most haze episodes in the winter in southeastern China usually lasted for 2-3 days. Therefore, 3-day haze exposure is representative of haze exposure.

After 3 days of haze exposure, all the mice in the three groups were exposed to filtered air to allow recovery for 7 days. To select the appropriate reasonable days, we previously treated mice with acute PM exposure by tracheal instillation and then traced the recovery process. In most cases, the mice with moderate PM exposure levels returned to their initial levels in 4 to 6 days. On this basis, we designed a 7-day recovery phase in this study. In addition, although most indexes of the LPM group recovered in 7 days, most indexes of the FA group remained at a high level at the end of the recovery phase. Therefore, in future experiments, observation of the recovery period could be further extended to further reveal the trend rules of the FA group.

**Sampling Site and PM_2.5_ Sample Preparation**

Nanjing (118°46′ E, 32°03′ N) is a megacity in southeastern China. It has a total area of approximately 6600 km^2^ and a population of over 8.1 million. It is one of the most important comprehensive industrial production bases and the main transportation hub in East China. Nanjing is located in a north subtropical monsoon climate zone. It has an annual mean temperature of 16°C and a mean annual precipitation of 1106 mm. The prevailing wind typically blows from the southeast in summer and from the northwest in winter.

The Xianlin campus of Nanjing University is near the northern industrial zones and includes several industrial point sources. The PM_2.5_ sampling site was set on the roof of the School of the Environment of Nanjing University. Each sample filter was conditioned for 48 h in a desiccator at 25°C and 40% relative humidity before and after sampling and then weighed to determine the mass of PM_2.5_. Samples were collected throughout the day (from 8 a.m. of one day to 8 a.m. of the next day) from December 9, 2017 to January 20, 2018.

**Selection of the Indicators Associated with Immune Regulation**

**The indicators of proinflammatory response.** The response to stimuli or infection includes the early and sequential release of “go signals,” such as proinflammatory cytokines, chemokines, and cell adhesion molecules, that collectively facilitate leukocyte migration into tissues^3^. Tumor necrosis factor α (*Tnf)*, interleukin 1b (*Il1b*), and *Il6*, three typical proinflammatory cytokines, were selected to follow the haze-induced proinflammatory response at the transcriptional and protein levels^4^. Chemokines and adhesion molecules are responsible for amplifying the inflammatory signal and recruiting leukocytes to the lesion site^5^. Thus, in this study, the transcript levels of three chemokines (*Ccl2, Ccl3*, and *Ccl5*) and three adhesion molecules (*Cd11c, Cc1d1*, and *Cd83*) were measured.

**The indicators of anti-inflammatory response.** Anti-inflammatory regulation is closely associated with the pro-inflammatory response and plays an important role in protecting the organism against an uncontrolled inflammatory burst. As an important anti-inflammatory cytokine, *Il10* inhibits microglial activation and limits the secretion of proinflammatory cytokines^6^. Cluster of differentiation 180 (*Cd180*) and leucine-rich repeat-containing protein 33 (*Nrros*) can negatively regulate Toll-like receptor (TLR) signaling, thus dampening TLR-driven proinflammatory events^7-9^. In this study, *Il10*, *Cd180* and *Nrros* were analyzed at the mRNA and protein levels as a measure of the anti-inflammatory response in mouse lung tissues.

**The indicators of inflammatory resolution.** The resolution of inflammation is indicated by a phase that begins with a peak in the release of inflammatory cells followed by their complete elimination and accompanied by the recovery of functional homeostasis. The dampening of proinflammatory signals and the elimination of inflammatory cells and damaged proteins are essential and crucial steps for the resolution process. Changes in the expression levels of steroid receptor coactivator 3 (*Rbms2*), zinc finger protein 36 (*Zfp36*), and zinc finger CCCH domain-containing protein 12 (*Zc3h12a*) are components of a regulatory mechanism that dampens proinflammatory signaling^10-12^. The clearance of apoptotic cells through efferocytosis is mediated by annexin A1 (*Anxa1*)-, signal-transducing adaptor molecule (*STAM*) binding protein (*Stambp*)-, and retinoid acid receptor responder 2 (*Rarres2*)-dependent signaling pathways. The proteasome is a multisubunit complex whose biological functions include the degradation of damaged proteins and the repair of DNA damage^13,14^. The transcriptional levels of two proteasomal subunits *(Psmb4* and *Psmd8*) and proteasomal activity were examined in the three groups of mice.

**The indicators of signaling pathway**. The cell cycle signaling pathway, which is indirectly involved in the inflammatory response, was also analyzed. The p53/p21 signaling pathway is the core signaling pathway regulating cell cycle arrest, and p38 MAPK is one of its multiple upstream signals activated by high intracellular levels of reactive oxygen species (ROS)^15^.

**Table S1.** Sequences of the primers used in the quantitative reverse transcription polymerase chain reaction.

| mRNA | Accession no. | Nucleotide sequence (5’→3’) | Size (bp) |
| --- | --- | --- | --- |
| *Anxa1* | NM_010730.2 | F:CCCCTACCCTTCCTTCAATGT | 269 |
|  |  | R:TGGCACCACGGAGTTCATCT |  |
| *Ccl2* | NM_002982.4 | F:TCCCAAAGAAGCTGTGATCTTCA | 104 |
|  |  | R:TTTGCTTGTCCAGGTGGTCC |  |
| *Ccl3* | NM_002983.3 | F:TTCCGTCACCTGCTCAGAAT | 103 |
|  |  | R:CAGCAGCAAGTGATGCAGAGA |  |
| *Ccl5* | NM_002985.3 | F:GGATCAAGACAGCACGTGGA | 248 |
|  |  | R:TCGGGTGACAAAGACGACTG |  |
| *Cd11c* | NM_025735.3 | F:ATCATCGAGCGCTACAAGGG | 262 |
|  |  | R:AGCCGAAGGTTTCTTGGGAG |  |
| *Cd1d1* | NM_007639.3 | F:TGGTGTGTCATGTCTCTGGC | 230 |
|  |  | R:CCTGGCATCCCAGTAGAGGA |  |
| *Cd83* | NM_004233.4 | F:TGCAACTCGGGGACATACAG | 205 |
|  |  | R:GCCGTGCAAACTTACAAGTGA |  |
| *Cd180* | NM_001360519.1 | F:GAATTGCCTTCTGGACTTGTGG | 142 |
|  |  | R:GTCTCTTCGTGTTGCCCTTGAT |  |
| *Il6* | NM_031168.1 | F:GAAATGATGGATGCTACCAAACTG | 140 |
|  |  | R:GACTCTGGCTTTGTCTTTCTTGTT |  |
| *Il1b* | NM_008361.4 | F:GGGCCTCAAAGGAAAGAATC | 183 |
|  |  | R:TACCAGTTGGGGAACTCTGC |  |
| *Il10* | NM_010548.2 | F:TTTAAGGGTTACTTGGGTTGCC | 106 |
|  |  | R:AATGCTCCTTGATTTCTGGGC |  |
| *Nrros* | NM_001347181.1 | F:TCTTGATTGTGGAATGGAGGAGC | 184 |
|  |  | R:TGGTTCCACAGGTCCTTGAGAG |  |
| *Psmb4* | NM_008945.3 | F:CTCGGCCAGATGGTGATT | 195 |
|  |  | R:ACGGGCATCTCGGTAGTA |  |
| *Psmd8* | NM_002812.4 | F:CTGGCCCGTGACATACTGGA | 246 |
|  |  | R:TTCCGGCTTCTGCTGCTG |  |
| *P38* | NM_001168508.1 | F:GTGCCCGAACGATACCAGAAC | 241 |
|  |  | R:TGAATTCCTCCAGTGACCTTGC |  |
| *P53* | NM_001127233.1 | F:ATGAACCGCCGACCTATCCT | 264 |
|  |  | R:GCGGATCTTGAGGGTGAAATAC |  |
| *P21* | NM_009429.3 | F:CAGCCATGACGAGCTGTTCT | 168 |
|  |  | R:CTTTCGGTACCTTCGCCCTC |  |
| *Rarres2* | NM_002889.4 | F:ATCGGTCGACGCATGAAGTGCTTGCTGATCTC | 203 |
|  |  | R:ATCGCTCGAGTTTGGTTCTCAGGGC |  |
| *Rbms2* | NM_001374779.1 | F:AGAGGCAGTGGGTACTAGGG | 143 |
|  |  | R:CCTAGAGGTCTGCTCCTGGT |  |
| *Stambp* | NM_001278581.1 | F:CCTGCTTCAGACCTCCATAGATG | 162 |
|  |  | R:AGGGGCTGTTCATCTCCGTT |  |
| *Tnf* | NM_008337.4 | F:CACCACCATCAAGGACTCAA | 251 |
|  |  | R:GAGACAGAGGCAACCTGACC |  |
| *Zfp36* | NM_003407.5 | F:ACTTCAGCGCTCCCACTCTC | 87 |
|  |  | R:GACAGGAGGCTCTCGTAGATG |  |
| *Zc3h12a* | NM_025079.3 | F:TCGTGGTTTCCAACGACACA | 118 |
|  |  | R:TCAGGGGGCATAAACTTGTCA |  |

**Table S2.** The mRNA expression levels of proinflammatory cytokines (*Tnf*, *Il1b*, *Il6*), chemokines (*Ccl2*, *Ccl3*, *Ccl5*) and adhesion molecules (*Cd11c*, *Cd1d1*, *Cd180*) at the termination of the preliminary exposure (PE) and haze challenge (HC) phases as well as on day 1 (FA1), day 3 (FA3), and day 7 (FA7) during the filtered air recovery phrase in the three groups with preliminary exposure to filtered air (FA), unfiltered air (UFA), or the air with a low PM concentration (LPM), respectively.

| Index | Groups | Time points | | | | |
| --- | --- | --- | --- | --- | --- | --- |
|  |  | PE | HC | FA1 | FA3 | FA7 |
| *Tnf* | FA group | 0.531±0.079a | 4.635±0.671c | 5.691±0.848c | 2.892±0.38b | 1.493±0.196b |
|  | LPM group | 0.681±0.060a | 3.892±0.384c | 2.093±0.385b | 1.060±0.128a | 0.776±0.092a |
|  | UFA group | 0.995±0.113a | 2.566±0.528bc | 2.962±0.419c | 2.341±0.395bc | 1.774±0.251b |
| *Il1b* | FA group | 0.816±0.167a | 6.894±0.976c | 5.643±0.916c | 4.053±0.488b | 2.373±0.428b |
|  | LPM group | 1.197±0.120a | 4.093±0.798c | 2.779±0.468bc | 1.541±0.209ab | 0.495±0.117a |
|  | UFA group | 1.013±0.145 | 1.872±0.325 | 1.372±0.253 | 1.517±0.286 | 1.443±0.226 |
| *Il6* | FA group | 0.424±0.141a | 5.279±0.830cd | 7.157±1.002d | 4.324±0.371bc | 3.034±0.225b |
|  | LPM group | 0.847±0.354a | 4.426±0.845c | 3.517±0.681bc | 2.082±0.487ab | 1.229±0.197a |
|  | UFA group | 1.004±0.128a | 1.578±0.310ab | 2.615±0.365c | 1.88±0.242bc | 2.031±0.224bc |
| *Ccl2* | FA group | 0.771±0.162a | 2.868±0.576bc | 6.785±1.120d | 4.533±0.425c | 1.257±0.129ab |
|  | LPM group | 0.885±0.221a | 5.049±1.034b | 2.197±0.386a | 1.348±0.166a | 0.625±0.118a |
|  | UFA group | 1.013±0.126 | 1.502±0.151 | 2.002±0.267 | 1.961±0.387 | 1.642±0.283 |
| *Ccl3* | FA group | 0.368±0.035a | 0.971±0.146b | 2.573±0.445c | 1.926±0.376c | 1.400±0.199bc |
|  | LPM group | 0.671±0.070a | 3.818±0.314c | 2.7±0.421b | 1.197±0.244a | 0.692±0.129a |
|  | UFA group | 0.993±0.118a | 1.738±0.263ab | 2.266±0.303b | 2.192±0.311b | 2.008±0.226b |
| *Ccl5* | FA group | 0.783±0.121a | 3.274±0.632b | 3.439±0.409b | 3.158±0.293b | 1.208±0.079a |
|  | LPM group | 0.595±0.102a | 3.891±0.696b | 2.787±0.408b | 1.153±0.230a | 0.782±0.071a |
|  | UFA group | 1.008±0.147 | 1.495±0.170b | 1.128±0.084a | 0.848±0.102a | 1.005±0.068a |
| *Cd11c* | FA group | 0.616±0.072a | 2.512±0.487bc | 3.211±0.473c | 3.240±0.271c | 2.034±0.300b |
|  | LPM group | 1.106±0.084a | 2.596±0.301b | 2.119±0.251b | 1.036±0.208a | 0.89±0.106a |
|  | UFA group | 1.012±0.077 | 0.938±0.055 | 0.849±0.166 | 1.173±0.199 | 1.085±0.144 |
| *Cd1d1* | FA group | 0.793±0.083a | 1.968±0.212c | 3.158±0.309d | 1.476±0.196bc | 0.861±0.076ab |
|  | LPM group | 0.933±0.187a | 2.205±0.370c | 1.726±0.163bc | 1.31±0.186ab | 1.297±0.120a |
|  | UFA group | 1.002±0.130 | 1.703±0.266 | 1.75±0.357 | 1.569±0.257 | 1.472±0.235 |
| *Cd83* | FA group | 0.329±0.057a | 3.902±0.680c | 3.999±0.501c | 2.065±0.349b | 1.905±0.383b |
|  | LPM group | 1.079±0.188a | 2.108±0.387b | 2.561±0.317b | 0.757±0.150a | 1.094±0.217a |
|  | UFA group | 1.007±0.114 | 0.905±0.116 | 0.964±0.130 | 0.844±0.091 | 1.210±0.169 |

*Note.* One-way ANOVA was used to determine differences across the other five time points (PE, HC, FA1, FA3 and FA7) in each group. P values less than 0.05 (P < 0.05) were considered significan

**Table S3.** Relative mRNA expression levels of anti-inflammatory factors (*Il10*, *Cd180*, *Nrros*) at the termination of the preliminary exposure (PE) and haze challenge (HC) phases as well as on day 1 (FA1), day 3 (FA3) and day 7 (FA7) during the filtered air recovery phase in the three groups with preliminary exposure to filtered air (FA), unfiltered air (UFA), or the air with a low PM concentration (LPM), respectively.

| Index | Groups | Time points | | | | |
| --- | --- | --- | --- | --- | --- | --- |
|  |  | PE | HC | FA1 | FA3 | FA7 |
| *Il10* | FA group | 0.501±0.083a | 0.828±0.152a | 1.129±0.28a | 4.965±0.979c | 3.126±0.385b |
|  | LPM group | 0.656±0.077a | 1.121±0.234a | 4.047±0.547c | 2.494±0.294b | 1.292±0.181ab |
|  | UFA group | 1.016±0.122a | 1.638±0.323ab | 2.261±0.419bc | 2.818±0.585c | 2.107±0.263b |
| *Cd180* | FA group | 0.688±0.111a | 0.736±0.140a | 0.705±0.217a | 1.342±0.288a | 5.609±0.840b |
|  | LPM group | 0.618±0.068a | 2.144±0.402b | 4.267±0.618c | 3.465±0.513c | 1.461±0.193ab |
|  | UFA group | 0.999±0.151 | 1.081±0.186 | 1.216±0.228 | 1.459±0.273 | 1.176±0.088 |
| *Nrros* | FA group | 1.227±0.122ab | 0.835±0.149a | 0.799±0.203a | 2.018±0.26c | 1.590±0.2870c |
|  | LPM group | 1.120±0.113a | 1.749±0.212a | 3.275±0.453b | 3.079±0.325b | 0.910±0.159a |
|  | UFA group | 0.997±0.118a | 1.152±0.154a | 1.495±0.219a | 2.230±0.306b | 1.676±0.235b |

*Note.* One-way ANOVA was used to determine differences across the other five time points (PE, HC, FA1, FA3 and FA7) in each group. P values less than 0.05 (P < 0.05) were considered significant.

**Table S4.** Relative mRNA expression levels of genes encoding proteins that dampen proinflammatory signaling (*Rbms2*, *Zfp36*, *Zc3h12a*), participate in efferocytosis (*Anxa1*, *Stambp*, *Rarres2*) and contribute to proteolytic systems (*PMSB4*, *PMSD8*) at the termination of the preliminary exposure (PE) and haze challenge (HC) phases as well as on day 1 (FA1), day 3 (FA3) and day 7 (FA7) during the filtered air recovery phase in the three groups with preliminary exposure to filtered air (FA), unfiltered air (UFA), or the air with a low PM concentration (LPM), respectively.

| Index | Groups | Time points | | | | |
| --- | --- | --- | --- | --- | --- | --- |
|  |  | PE | HC | FA1 | FA3 | FA7 |
| *Rbms2* | FA group | 0.962±0.103a | 0.670±0.182a | 0.664±0.121a | 2.979±0.401c | 2.092±0.242b |
|  | LPM group | 0.649±0.130a | 0.587±0.163a | 1.934±0.391b | 1.354±0.262ab | 1.107±0.255a |
|  | UFA group | 1.002±0.177 | 0.533±0.061 | 0.851±0.162 | 0.914±0.195 | 0.842±0.111 |
| *Zfp36* | FA group | 1.961±0.370b | 0.948±0.189a | 0.461±0.091a | 1.249±0.200ab | 2.893±0.461b |
|  | LPM group | 2.243±0.135a | 2.452±0.427a | 2.878±0.565a | 4.814±0.804b | 1.822±0.191a |
|  | UFA group | 1.011±0.101ab | 0.605±0.075a | 0.766±0.154ab | 1.129±0.143b | 1.202±0.196b |
| *Zc3h12a* | FA group | 1.252±0.168ab | 1.450±0.296ab | 0.884±0.151a | 3.707±0.446c | 1.874±0.351b |
|  | LPM group | 1.501±0.077a | 2.625±0.354b | 2.744±0.427b | 1.497±0.251a | 1.044±0.195a |
|  | UFA group | 1.008±0.122 | 1.264±0.168 | 1.399±0.186 | 1.567±0.284 | 1.442±0.204 |
| *Anxa1* | FA group | 1.696±0.175a | 1.292±0.18a | 2.097±0.356a | 4.706±0.644b | 6.799±1.238c |
|  | LPM group | 2.739±0.504a | 2.530±0.226a | 6.900±1.296b | 9.099±1.900b | 2.501±0.263a |
|  | UFA group | 0.996±0.152a | 0.602±0.026b | 0.455±0.033a | 0.658±0.125a | 0.685±0.064a |
| *Stambp* | FA group | 2.406±0.323a | 1.093±0.117a | 1.697±0.236ab | 6.202±1.019b | 12.008±2.885c |
|  | LPM group | 3.006±0.374ab | 1.998±0.359a | 7.997±1.338c | 6.006±1.524b | 2.491±0.586a |
|  | UFA group | 1.016±0.078 | 1.115±0.171 | 1.198±0.146 | 1.241±0.180 | 1.031±0.139 |
| *Rarres2* | FA group | 1.399±0.166a | 1.676±0.259ab | 1.800±0.326ab | 3.69±0.492c | 2.697±0.386bc |
|  | LPM group | 1.861±0.255a | 2.431±0.463a | 5.087±0.685b | 4.619±0.601b | 2.552±0.428a |
|  | UFA group | 1.003±0.189 | 0.855±0.273 | 0.576±0.055 | 0.651±0.069 | 0.776±0.152 |
| *Psmb4* | FA group | 3.539±0.133d | 11.186±0.696b | 2.513±0.256a | 10.86±0.588b | 24.70±0.981c |
|  | LPM group | 4.311±0.086a | 3.957±0.483b | 11.184±0.756d | 9.550±0.395c | 3.210±0.135a |
|  | UFA group | 1.000±0.125 | 0.897±0.129a | 1.471±0.075ab | 2.302±0.132b | 1.436±0.076ab |
| *Psmd8* | FA group | 5.221±0.137d | 8.299±0.498b | 1.771±0.220a | 10.111±0.572b | 20.377±0.747c |
|  | LPM group | 4.533±0.169a | 4.348±0.365a | 16.187±0.173b | 7.381±0.464a | 2.721±0.184a |
|  | UFA group | 1.003±0.088 | 0.589±0.147a | 1.108±0.136a | 1.714±0.118a | 1.318±0.116b |

*Note.* One-way ANOVA was used to determine differences across the other five time points (PE, HC, FA1, FA3 and FA7) in each group. P values less than 0.05 (P < 0.05) were considered significant.

**Table S5.** Relative levels of *P38*, *P53* and *P21* mRNA expression at the termination of the preliminary exposure (PE) and haze exposure (HE) phases in the three groups with preliminary exposure to filtered air (FA), unfiltered air (UFA), or the air with a low PM concentration (LPM), respectively.

| Groups | *P38* | |  | *P53* | |  | *P21* | |
| --- | --- | --- | --- | --- | --- | --- | --- | --- |
|  | PE | HC |  | PE | HC |  | PE | HC |
| FA group | 4.058±0.215 | 8.125±1.105 |  | 1.163±0.069 | 1.444±0.145 |  | 0.278±0.011 | 1.456±0.076 |
| LPM group | 1.747±0.145 | 2.900±0.363 |  | 1.174±0.074 | 1.032±0.135 |  | 0.538±0.035 | 0.468±0.031 |
| UFA group | 1.000±0.113 | 1.542±0.197 |  | 1.000±0.057 | 1.564±0.077 |  | 1.00±0.0471 | 0.903±0.068 |


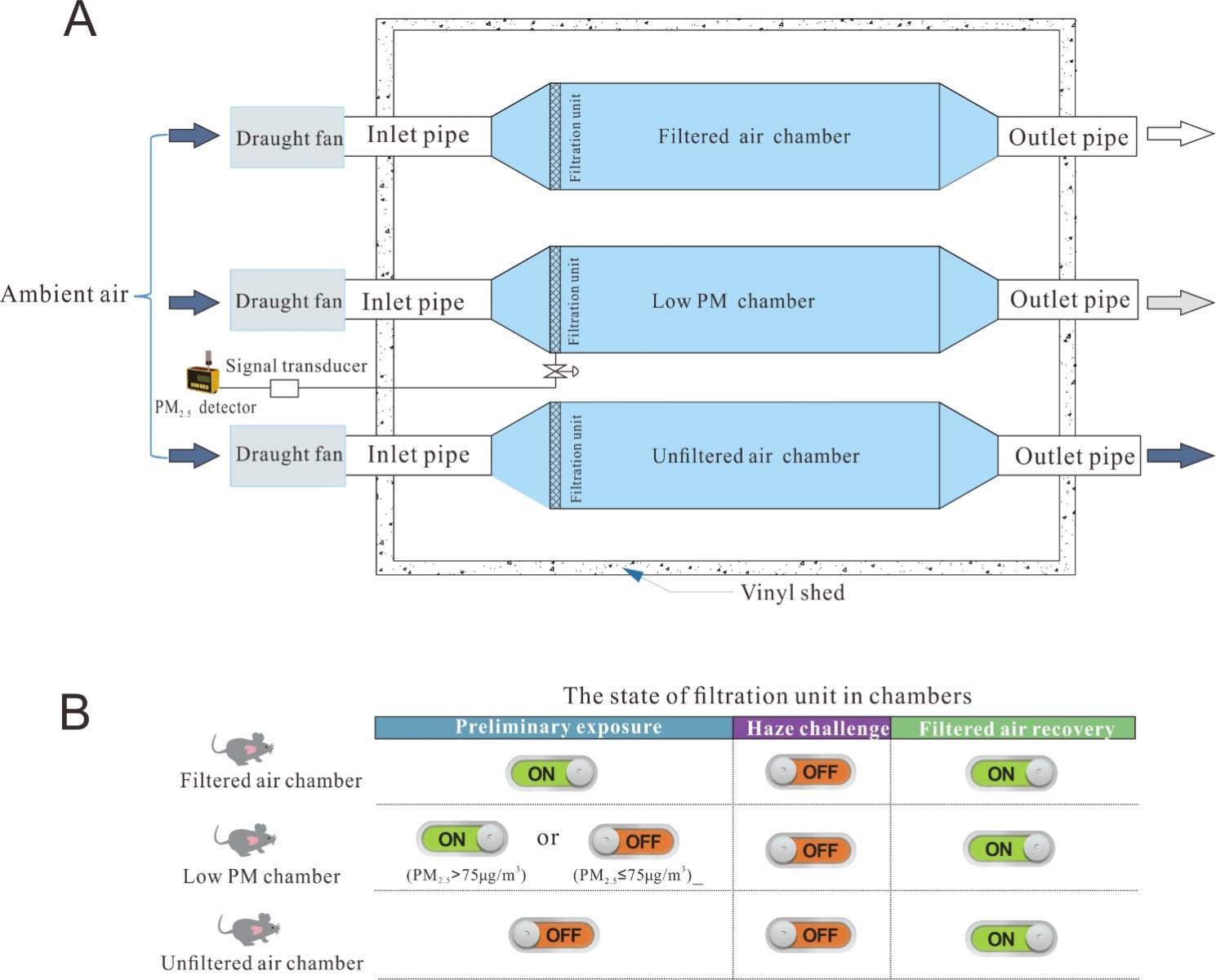


**Figure S1.** Scheme for the operating principle of the exposure chamber. The mice were raised in exposure chambers placed separately in vinyl sheds. (A) A draught fan placed outdoors was linked to each exposure chamber to allow the ambient air to flow in. A PM filtration unit was fixed between the air inlet and exposure space in each chamber. (B) Ambient or filtered air was delivered to each exposure chamber by controlling the on/off state of the air filtration unit.


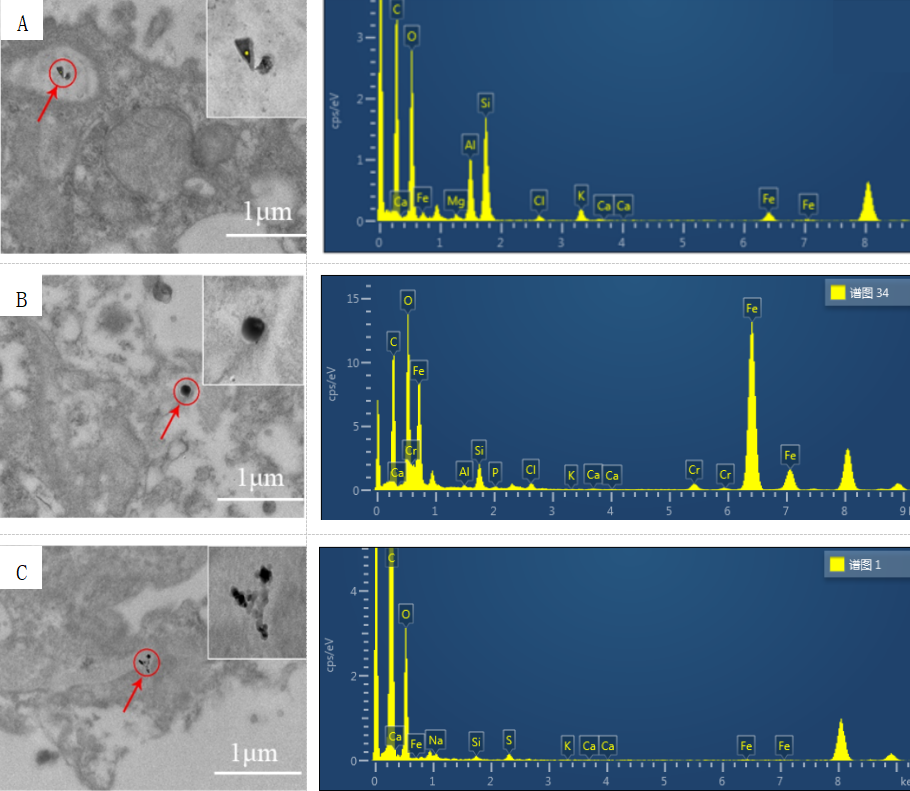


**Figure S2.** The combined high-resolution transmission electron micrographs and EDS analysis of the lungs of mice in the unfiltered group revealed three distinct types of PM. (A) Polygonal, angular particles containing high levels of silicon (Si), oxygen(O), aluminum (Al), etc., consistent with their MD origin^16^, (B) roughly spherical particles containing heavy metals such as iron (Fe) and chromium (Cr), suggesting the high-temperature formation of nanospheres via combustion and/or friction^17^ and (C) aggregate carbon particles containing high contents of carbon (C) and oxygen (O), identified the presence of fly ash from industrial coal combustion^16^.


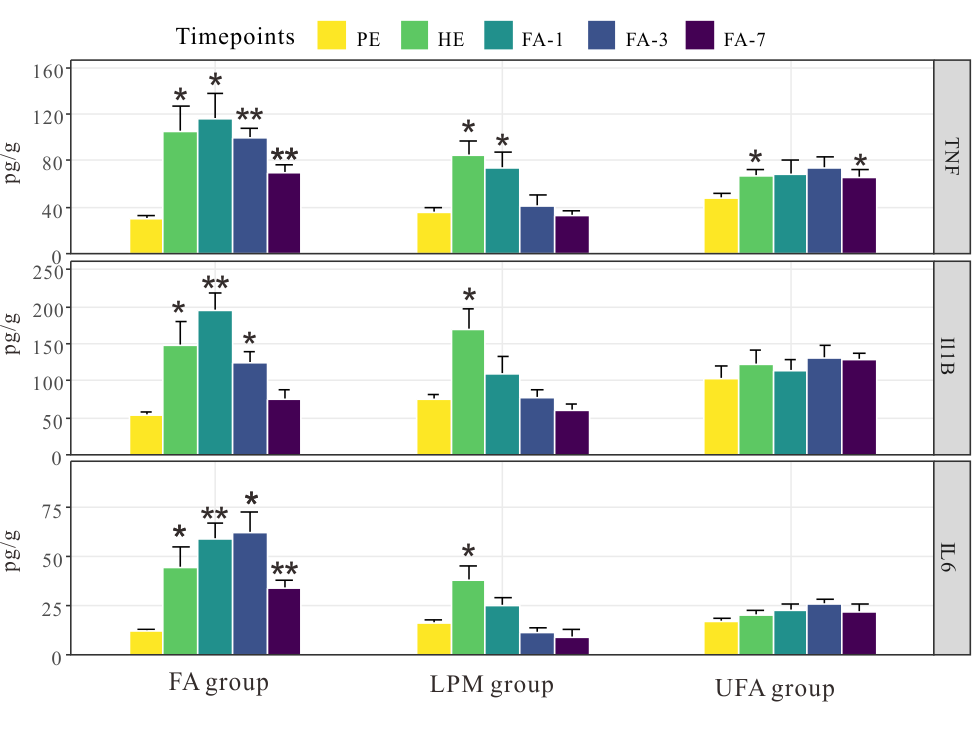


**Figure S3.** The protein levels of TNF, IL1B, and IL6 in mouse lungs were measured at the termination of the preliminary exposure (PE) and haze challenge (HC) phases as well as on day 1 (FA1), day 3 (FA3) and day 7 (FA7) during the filtered air recovery phase in the three groups with preliminary exposure to filtered air (FA), unfiltered air (UFA), or the air with a low PM concentration (LPM), respectively. The bars indicate the error of the mean (n = 5-7); *p < 0.05 and **p < 0.0, compared with the PE time point in a One-way ANOVA followed by Tukey's post-hoc test.

.


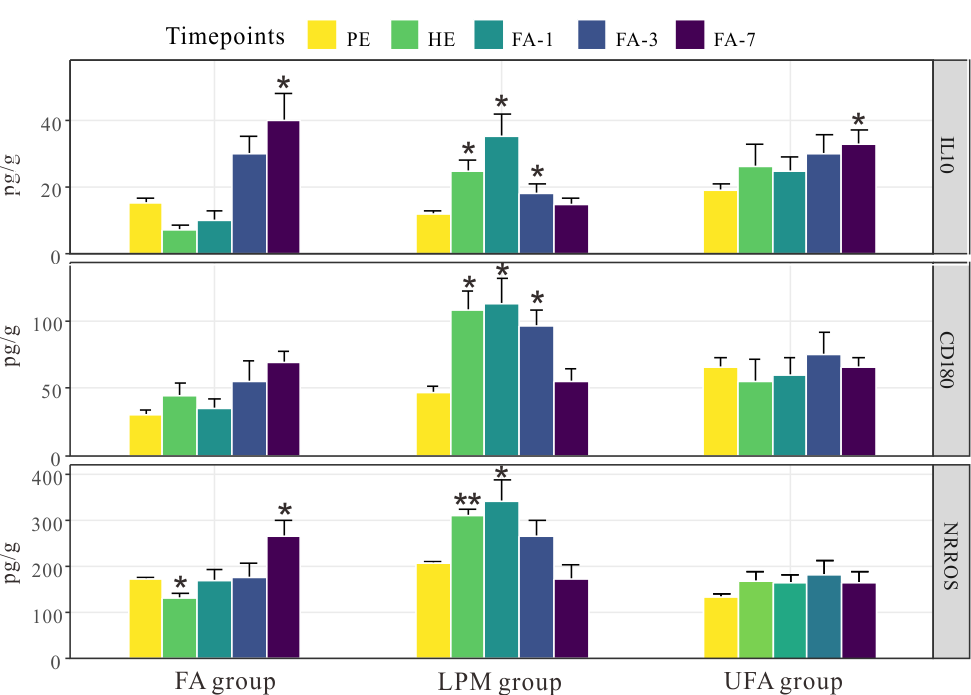


**Figure S4.** IL10, CD180, and NRROS in mouse lungs protein levels were assayed at the termination of preliminary exposure (PE) and haze challenge (HC) phases as well as on day 1 (FA1), day 3 (FA3) and day 7 (FA7) during the filtered air recovery phase in the three groups with preliminary exposure to filtered air (FA), unfiltered air (UFA), or the air with a low PM concentration (LPM), respectively. The bars indicate the standard error of the mean (n = 5-7); *p < 0.05 and **p < 0.01 compared with the PE time point in a One-way ANOVA followed by Tukey's post-hoc test.


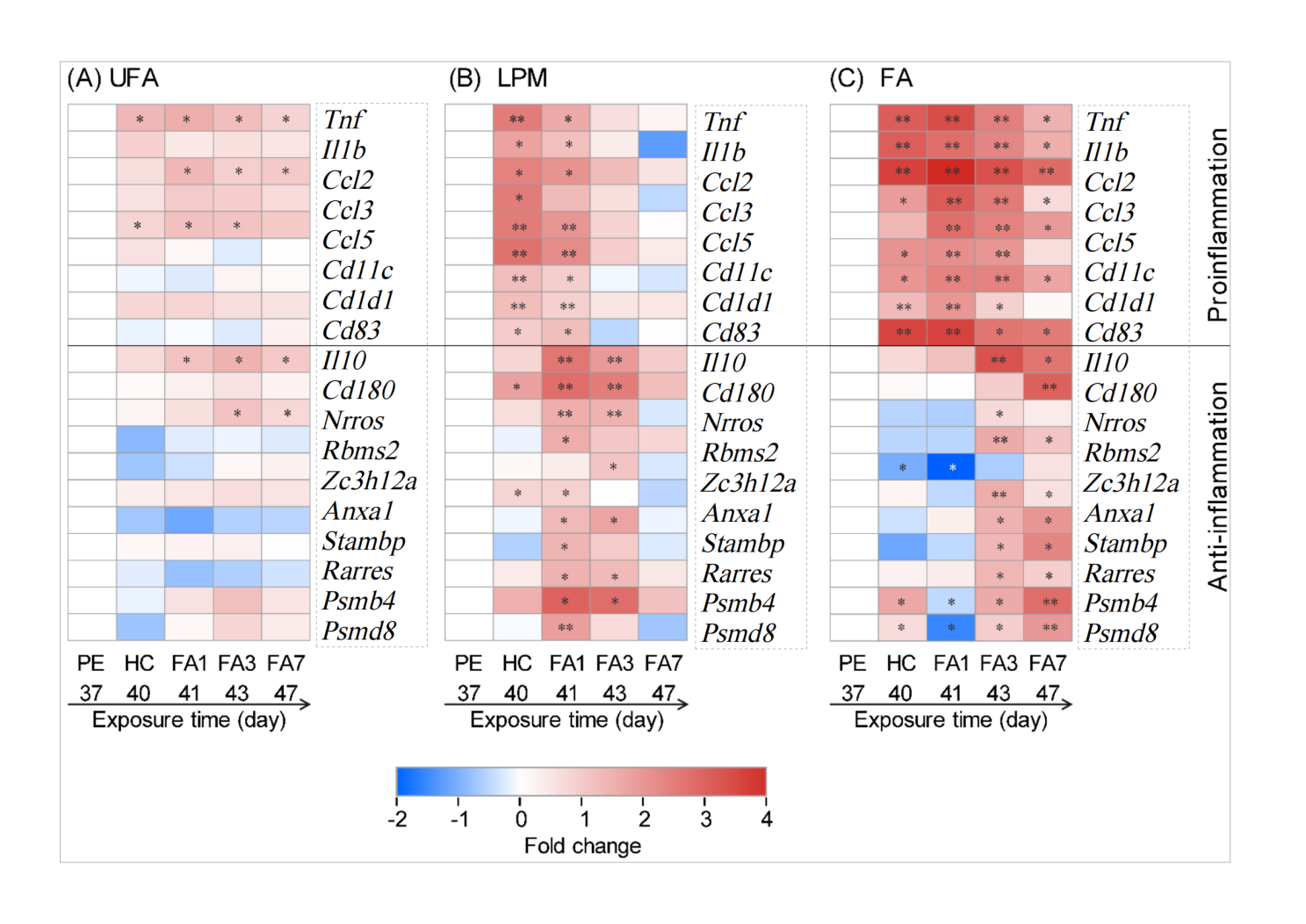


**Figure S5.** Summarized relative expression level of proinflammatory and anti-inflammatory genes in mouse lungs upon PM exposure. (A) Pre-exposure (PE) with unfiltered air (UFA) followed by haze challenge (HZ) and filtered air (FA) exposure. (B) PE with low PM concentration air (LPM) followed by HZ and FA exposure. (C) PE with FA followed by HZ and FA exposure. The relative expression of each gene at HC and followed FA (FA1, 3, 7) were presented with respect to its expression level at the termination of PE. Relative expression were presented as Log2 fold change against PE treatment. p < 0.05 and **p < 0.01 compared with the PE time point in a One-way ANOVA followed by Tukey's post-hoc test.


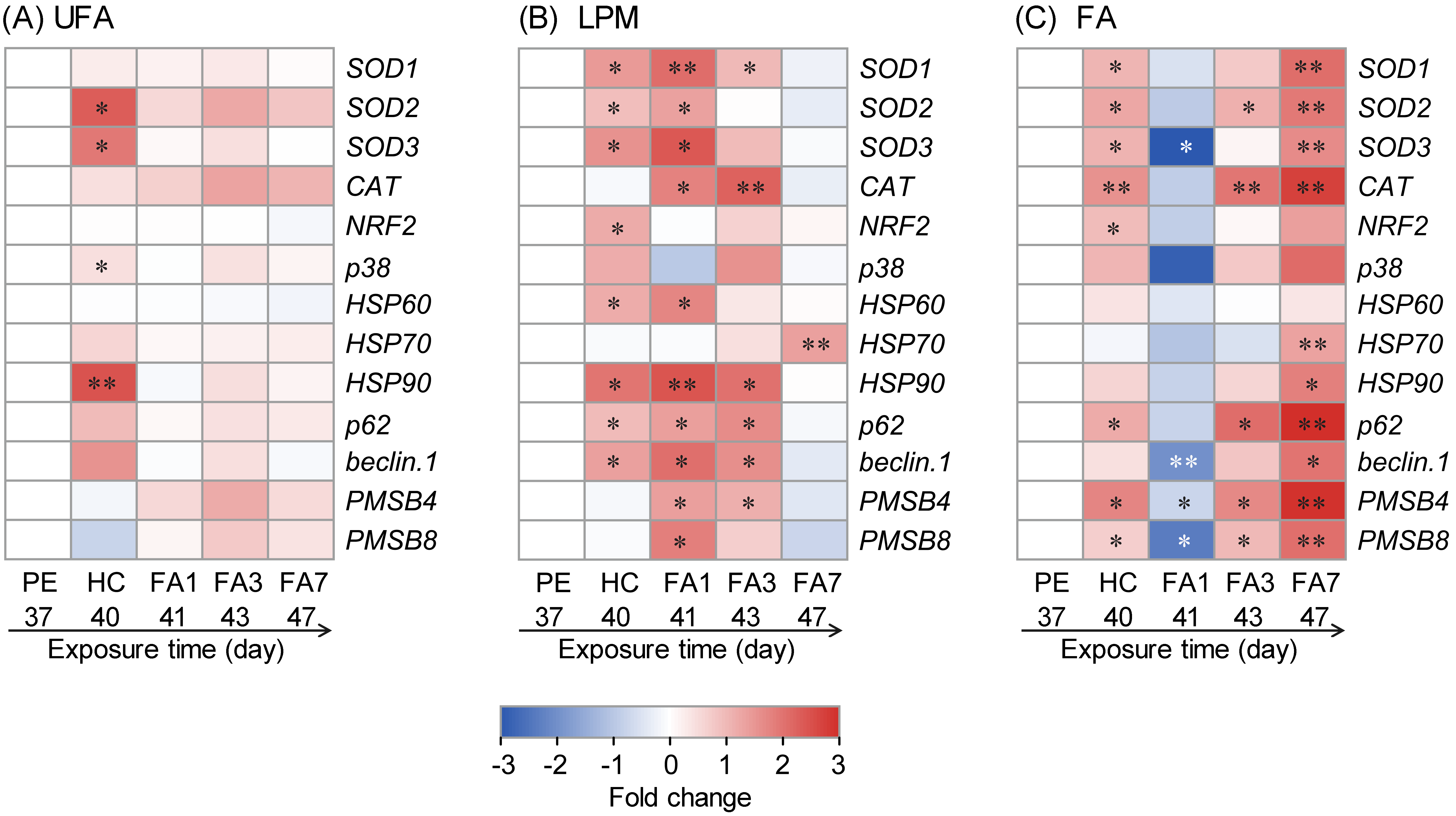


**Figure S6.** Summarized relative expression level of antioxidant-related genes genes in mouse lungs upon PM exposure. (A) Pre-exposure (PE) with unfiltered air (UFA) followed by haze challenge (HZ) and filtered air (FA) exposure. (B) PE with low PM concentration air (LPM) followed by HZ and FA exposure. (C) PE with FA followed by HZ and FA exposure. The relative expression of each gene at HC and followed FA (FA1, 3, 7) were presented with respect to its expression level at the termination of PE. Relative expression were presented as Log2 fold change against PE treatment. p < 0.05 and **p < 0.01 compared with the PE time point in a One-way ANOVA followed by Tukey's post-hoc test.

**References**

1. Liu, X. *et al.* Particulate Matter Exposure History Affects Antioxidant Defense Response of Mouse Lung to Haze Episodes. *Environmental Science & Technology* **53**, 9789-9799, doi:10.1021/acs.est.9b01068 (2019).

2. Liu, X. *et al.* Particulate matter exposure disturbs inflammatory cytokine homeostasis associated with changes in trace metal levels in mouse organs. *Science of the Total Environment* **727**, doi:10.1016/j.scitotenv.2020.138377 (2020).

3. Fullerton, J. N. & Gilroy, D. W. Resolution of inflammation: a new therapeutic frontier. *Nat. Rev. Drug Discovery* **15**, 551-567, doi:10.1038/nrd.2016.39 (2016).

4. Kany, S., Vollrath, J. T. & Relja, B. Cytokines in Inflammatory Disease. *International Journal of Molecular Sciences* **20**, doi:10.3390/ijms20236008 (2019).

5. Hotamisligil, G. S. Foundations of Immunometabolism and Implications for Metabolic Health and Disease. *Immunity* **47**, 406-420, doi:10.1016/j.immuni.2017.08.009 (2017).

6. Pan, Y. *et al.* The effects of central pro-and anti-inflammatory immune challenges on depressive-like behavior induced by chronic forced swim stress in rats. *Behav. Brain Res.* **247**, 232-240, doi:10.1016/j.bbr.2013.03.031 (2013).

7. Divanovic, S. *et al.* Negative regulation of Toll-like receptor 4 signaling by the Toll-like receptor homolog RP105. *Nat. Immunol.* **6**, 571-578, doi:10.1038/ni1198 (2005).

8. Liu, J. *et al.* Identification and characterization of a unique leucine-rich repeat protein (LRRC33) that inhibits Toll-like receptor-mediated NF-kappa B activation. *Biochem. Biophys. Res. Commun.* **434**, 28-34, doi:10.1016/j.bbrc.2013.03.071 (2013).

9. van Strien, M. E. *et al.* Anti-inflammatory effect by lentiviral-mediated overexpression of IL-10 or IL-1 receptor antagonist in rat glial cells and macrophages. *Gene Ther.* **17**, 662-671, doi:10.1038/gt.2010.8 (2010).

10. Garcia-Maurino, S. M. *et al.* RNA Binding Protein Regulation and Cross-Talk in the Control of AU-rich mRNA Fate. *Frontiers in Molecular Biosciences* **4**, doi:10.3389/fmolb.2017.00071 (2017).

11. Lin, W. *et al.* Regulation of Nrf2 transactivation domain activity by p160 RAC3/SRC3 and other nuclear co-regulators. *J. Biochem. Mol. Biol.* **39**, 304-310 (2006).

12. Matsushita, K. *et al.* Zc3h12a is an RNase essential for controlling immune responses by regulating mRNA decay. *Nature* **458**, 1185-U1124, doi:10.1038/nature07924 (2009).

13. Pickering, Andrew M. *et al.* The immunoproteasome, the 20S proteasome and the PA28αβ proteasome regulator are oxidative-stress-adaptive proteolytic complexes. *Biochem. J.* **432**, 585-595, doi:10.1042/bj20100878 (2010).

14. Pickering, A. M., Linder, R. A., Zhang, H., Forman, H. J. & Davies, K. J. A. Nrf2-dependent Induction of Proteasome and Pa28αβ Regulator Are Required for Adaptation to Oxidative Stress*. *J. Biol. Chem.* **287**, 10021-10031, doi:https://doi.org/10.1074/jbc.M111.277145 (2012).

15. Munoz-Espin, D. & Serrano, M. Cellular senescence: from physiology to pathology. *Nature Reviews Molecular Cell Biology* **15**, 482-496, doi:10.1038/nrm3823 (2014).

16. Li, H. *et al.* Fractionation of airborne particulate-bound elements in haze-fog episode and associated health risks in a megacity of southeast China. *Environmental Pollution* **208**, 655-662, doi:10.1016/j.envpol.2015.10.042 (2016).

17. Maher, B. A. *et al.* Magnetite pollution nanoparticles in the human brain. *Proc. Natl. Acad. Sci. U. S. A.* **113**, 10797-10801, doi:10.1073/pnas.1605941113 (2016).
